# Genotype-Based Test in Mapping *Cis*-Regulatory Variants from Allele-Specific Expression Data

**DOI:** 10.1371/journal.pone.0038667

**Published:** 2012-06-07

**Authors:** Jean Francois Lefebvre, Emilio Vello, Bing Ge, Stephen B. Montgomery, Emmanouil T. Dermitzakis, Tomi Pastinen, Damian Labuda

**Affiliations:** 1 Centre de Recherche du CHU Sainte-Justine, Université de Montréal, Montréal, Québec, Canada; 2 McGill University and Genome Québec Innovation Centre, Montréal, Québec, Canada; 3 Department of Genetic Medicine and Development, University of Geneva Medical School, Geneva, Switzerland; 4 Wellcome Trust Sanger Institute, Cambridge, United Kingdom; 5 Department of Human Genetics, McGill University Health Centre, McGill University, Montréal, Québec, Canada; 6 Department of Medical Genetics, McGill University Health Centre, McGill University, Montréal, Québec, Canada; 7 Département de Pédiatrie, Université de Montréal, Montréal, Québec, Canada; Queen's University Belfast, United Kingdom

## Abstract

Identifying and understanding the impact of gene regulatory variation is of considerable importance in evolutionary and medical genetics; such variants are thought to be responsible for human-specific adaptation [1] and to have an important role in genetic disease. Regulatory variation in *cis* is readily detected in individuals showing uneven expression of a transcript from its two allelic copies, an observation referred to as allelic imbalance (AI). Identifying individuals exhibiting AI allows mapping of regulatory DNA regions and the potential to identify the underlying causal genetic variant(s). However, existing mapping methods require knowledge of the haplotypes, which make them sensitive to phasing errors. In this study, we introduce a genotype-based mapping test that does not require haplotype-phase inference to locate regulatory regions. The test relies on partitioning genotypes of individuals exhibiting AI and those not expressing AI in a 2×3 contingency table. The performance of this test to detect linkage disequilibrium (LD) between a potential regulatory site and a SNP located in this region was examined by analyzing the simulated and the empirical AI datasets. In simulation experiments, the genotype-based test outperforms the haplotype-based tests with the increasing distance separating the regulatory region from its regulated transcript. The genotype-based test performed equally well with the experimental AI datasets, either from genome–wide cDNA hybridization arrays or from RNA sequencing. By avoiding the need of haplotype inference, the genotype-based test will suit AI analyses in population samples of unknown haplotype structure and will additionally facilitate the identification of *cis*-regulatory variants that are located far away from the regulated transcript.

## Introduction

Genetic mechanisms that modulate gene expression contribute to human phenotypic variation and disease susceptibility [2]. Identifying regulatory elements that control RNA transcription efficiency or level is therefore of major general and medical interest [3]. Numerous investigations have contributed to the identification of putative regulatory variants [4], [5]. When these variants are located on the same chromosome as the transcript they regulate, they are expected to lead to allele-specific differences in the expression level of cognate transcripts. The resulting allele-specific expression (ASE) can be identified in heterozygous individuals that differentially express the two parental copies of the regulated transcript, also referred to as allelic imbalance (AI). Multiple efforts have been made to detect genes exhibiting ASE [6]–[9]. Less attention has been given to the mapping of regulatory elements and finding the underlying AI-causing regulatory variants [6], [7]. Working on small genetic distances facilitated the application of haplotype-based tests, especially when using cell lines of the HapMap project [10], [11] where chromosomal phasing, based on family trios is relatively reliable. However, phase uncertainty will be greater in populations of less well characterized haplotype structure, thus reducing the power of haplotype-based tests. Also, phasing accuracy decreases with an increasing genetic distance, hence the detection rate of regulatory variants that are located far away from their regulated transcripts can be particularly affected [5], [12]–[14]. In order to improve the mapping efficiency of regulatory elements and variants using AI data, we propose a genotype-based contingency test that is insensitive to phasing errors and can be applied genome-wide to map cis-regulatory variants. We compared this test with a standard linear regression test used by Ge et al. [6]and with another haplotype-based binomial test introduced here. We studied the performance of these tests in mapping regulatory elements in genes known to exhibit AI and where AI expressing individuals were already ascertained. Toward this end we used computer simulated data as well as empirical datasets of Ge et al. [6], and Montgomery et al. [7].

## Results

### Modeling linkage disequilibrium between regulatory elements and genes with allele-specific expression

**Figure 1:**
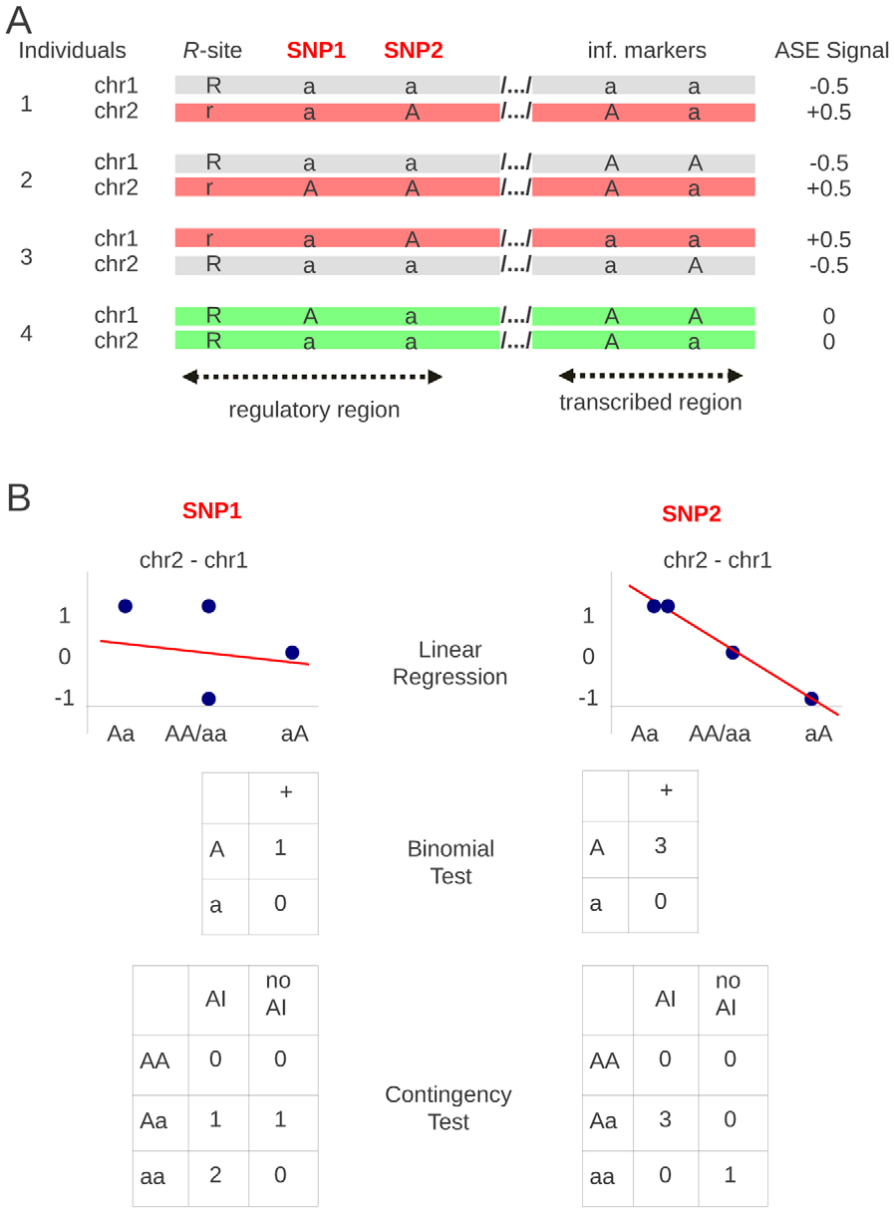
Ideograms of linear regression and binomial haplotype-based tests, and of contingency genotype-based test. How AI results are used in the three tests with hypothetical SNPs, SNP1 and SNP2, chosen such that SNP1 is not linked to the R-site whereas SNP2 is.

Consider a regulatory site *R* that affects expression of a gene X (Figure 1A). Of its two alleles R (ancestral) and r (derived), one causes up-regulation and the other down-regulation of the regulated transcript. As a consequence, RNAs transcribed from two parental copies of this gene are unequally expressed in Rr heterozygotes, causing AI that can be revealed by measuring relative levels of the corresponding allelic transcripts. On the chromosome expressing gene X, there are SNPs (referred to as sites *A*) that can be tested for association with AI caused by the *R* site. In Figure 1A, some of these sites (SNP1 and SNP2) are found within the regulatory region, in the vicinity and in linkage with the *R* site. Polymorphic sites that are found within the transcript itself are used as informative markers, which allow distinguishing between allelic transcripts from the two copies of the chromosome and their expression levels. In this example, there is no linkage between the regulatory region and the transcribed region polymorphisms. Informative markers instrumental in revealing AI and those that are informative in locating the *R* site are physically separated. This emphasizes the difference between the AI detection and the mapping of the corresponding regulatory region. In practice the majority of *cis-*regulatory elements are very close to the transcript they control, and tightly linked to the informative markers.

**Figure 2:**
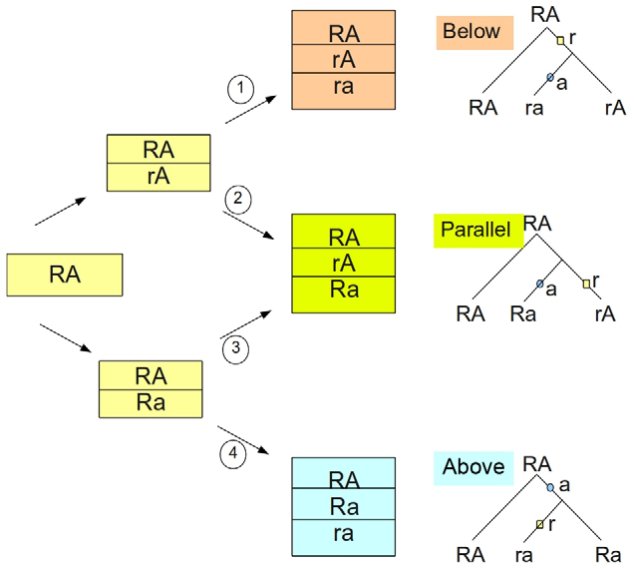
Four possible mutational pathways creating three distinct sets of three haplotypes. Depending on the sequence of mutations starting with the ancestral haplotype on the left, we obtain three sets of haplotypes, referred to as below, parallel and above to reflect the position of the *A*-site vs. *R*-site mutation on the genealogy shown on the right. These genealogical positions can be modified by recombination. We assume no recurrent mutations.

**Figure 3:**
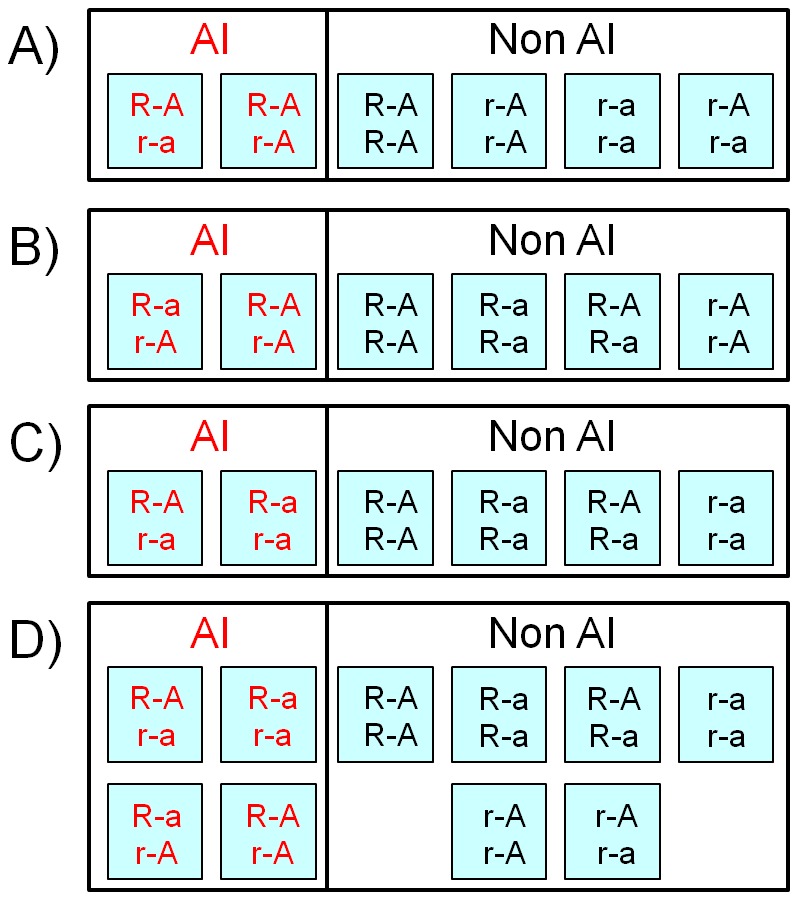
Sets of possible genotypes under complete and incomplete linkage disequilibrium. Under complete LD for genealogical positions below (**A**), parallel (**B**) and above (**C**), there are always two genotypes characterizing AI-individuals and only one type of A-site homozygote present (AA or aa). Under equilibrium or incomplete linkage disequilibrium (**D**) all four haplotypes involving R and A sites are present and thus potentially all ten resulting genotypes as well.

When site *R* and any of the tested SNPs (*A* sites) are unlinked, their respective alleles will segregate randomly. In contrast, SNPs located in the vicinity of the *R* site, in the absence of recombination, i.e. at complete LD between these two sites, co-segregate in a characteristic fashion. With two bi-allelic sites, there are four possible mutation histories, each one leading to a characteristic haplotype trio, i.e. to a combination of three possible haplotypes depending on the tree genealogy (Figure 2). The sites are referred to be in “parallel” position when a and r mutations originate on different branches; then both derived alleles, a and r, will occur on different haplotypes. The *A* site mutation and the *R* site mutation sequentially occurred on the same branch of the genealogy, with *A* site mutating first (thus referred to as “above”) or second (“below”). Mutation histories are mutually exclusive, yet histories 2 and 3, when the sites are in parallel position, are indistinguishable at the level of haplotype trios (Figure 2). From each haplotype trio, six different sets of diploid genotypes involving two bi-allelic sites, *A* and *R*, can potentially arise (Figure 3). In each set we find two genotypes representing Rr individuals that express AI phenotype. Importantly, in each of these sets the distribution of the *A* site genotypes differ between AI expressing individuals (Rr) and non-AI individuals (RR and rr).

### Haplotype-based tests

Observing the AI phenotype reveals the heterozygous status of the *R* site. R and r alleles are associated with either up- or down-regulation of transcription. Provided that haplotype phase is known, two different alleles of any heterozygous SNP from the same chromosome can be assigned to its up- or down regulated copy (Figure 1A). In the absence of LD between the *R* site and the genotyped SNPs, their A and a alleles are expected to be distributed with equal probability between up- and down regulated chromosomes in all AI individuals. In contrast, when an analyzed SNP is linked to the *R* site, its A or a allele will tend to be exclusively associated with only down- or up-regulated chromosomes. The significance of LD between the *R* site and a given SNP can be evaluated as binomial probability *p* of observing the data, assuming equal probability of the occurrence of each of the alleles on up- and down regulated chromosomes (Figure 1B). Obviously, this test only makes use of AI-(Rr) individuals that, in the same time, are Aa heterozygotes.

The second haplotype-based test is a linear regression test used by Ge at al. [6]. It consists of fitting a linear model through the observed individual AI intensities ordered by the genotype state of the analyzed SNP. AI intensity is measured as a difference between transcription levels from two parental copies, chromosome 1 and 2, arbitrarily numbered as they appear in the database. The genotype state represents the allelic status of these copies, such that “Aa” means ‘A’ on chromosome 2 and ‘a’ on chromosome 1, which is different than “aA”. Measures of transcription levels can vary substantially from one experiment to the other, which can directly alter linear regression significance. Using a simple rule explained in the Methods section, we first analyse ASE results to identify AI individuals and non-AI individuals. Then, by definition, the AI intensity of AI expressing individuals is either +1 or −1, and zero in non-AI individuals (Figure 1B). It usually leads to higher log(1/p) and to lower FPR of linear regression test as compared to how it is used in Ge et al. [6] where AI intensities are those measured directly and may differ among individuals.

### Genotype-based test

As shown in Figure 3, when *R* and *A* sites are in LD, each haplotype trio leads to a specific set of diploid genotypes where only one type of *A*-site homozygote, AA or aa, is expected to be observed in AI individuals (Rr). In contrast, in linkage equilibrium between different SNPs and the *R* site, homozygotes AA and aa as well as heterozygotes Aa are expected to be distributed with equal probability between AI and non-AI individuals. Therefore, deviation from random distribution of these genotypes using 2×3 contingency table (Fisher's exact–test) will indicate LD between this A-site and the *R* site (Figure 1B). In the setting of genetic association studies of complex phenotypes, this test is usually referred to as the two degrees-of-freedom genotypic test, and is already implemented in genetic statistical software such as PLINK [15].

### Performance of mapping tests in simulation experiments

The mapping potential of the proposed tests was studied through simulation experiments. We simulated DNA segments considering a range of allele frequencies at the alleged regulatory sites in a population of constant size with and without recombination. Recombination events were either distributed evenly or were concentrated in recombination hotspots. For simplicity, we only report the results considering SNPs with minor allele frequency (MAF) of 5% or more, which mimic empirical results using HapMap genotypes [10], [11]. Table 1 presents the simulation results of power estimates and false positive rates (FPR) of the three tests. Because FPR is highest in the absence of recombination, it is only reported for simulation experiments under this condition. In the three tests considered (Figure 1B) both power and FPR show overall dependence upon the frequency of the r-allele and the *R* site heterozygosity (Table 1). In the case of haplotype-based tests, power is positively correlated with *R* heterozygosity (i.e. number of AI individuals), which is maximal at the r-allele frequency of 0.5. In the case of the contingency test, the highest power is observed at the r-allele frequency of 0.85, thus correlating with the age of the regulatory mutation reflected in the frequency of the derived allele. However, in contrast to the contingency test, the power of haplotype-based tests is reduced due to phasing errors. This effect is non-negligible: after rephasing using fastPhase [16] we lost 13% to 29% of potentially significant SNPs in simulation experiments reported in Table 1. There are additional issues to be considered when evaluating the performance of these tests. The first concerns the spectra of possible *p*-values associated with each of these tests, how these reflect the extent of LD of the corresponding SNPs with the *R* site and, in the case of haplotype based tests, how these are affected by chromosome phasing as a function of genetic distance between the regulatory and the transcribed sequence. The second concerns the distribution of significant SNPs around the *R* site, how close they occur and the proportion of significant SNPs of poor “mapping value”.

**Table 1:** Power and False Positive Rate (FPR) in simulation experiments.

	Power (%)						FPR (%)					
	No Recombination				Recombination				No Recombination			
r frequency	0.15	0.35	0.5	0.85	0.15	0.35	0.5	0.85	0.15	0.35	0.5	0.85
Test												
Binomial	25	46	55	42	11	22	24	14	<0.1	0.1	0.4	<0.1
Contingency	27	32	31	53	16	12	9	19	0.6	0.8	0.7	<0.1
Linear Regression	35	55	67	59	23	32	35	28	1.2	1.0	1.1	<0.1

Note: Power was evaluated as the fraction of simulated SNPs (*A* sites) showing *p*-values<0.01 over all SNPs (only sites with MAF≥5% were considered). FPR was estimated by assigning AI status to randomly chosen individuals corresponding to the expected number of Rr heterozygotes,12, 22, 25 and 13, given r frequencies of 0.15, 0.35, 0.5 and 0.85, respectively. FPR is only reported in the case of no recombination, as it is smaller in the presence of recombination.

**Figure 4:**
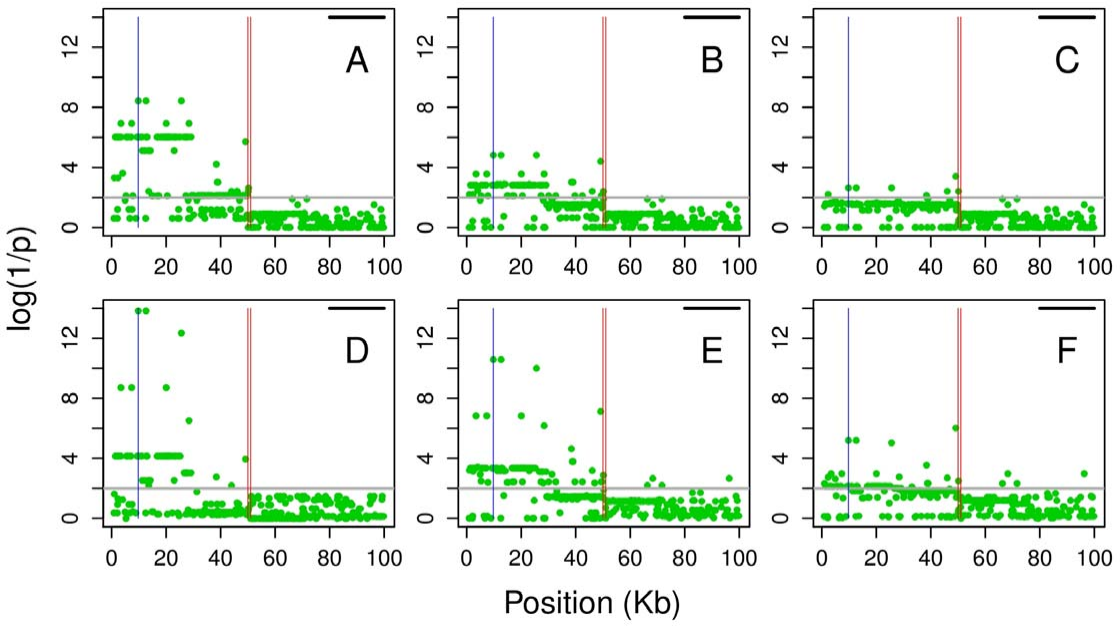
Looking for regulatory segment ∼0.1 cM from its regulated transcript. Vertical red lines in the middle correspond to the location of the recombination hotspot, the blue line on the left indicate the location of the *R* site, and the horizontal black line on the upper right corresponds to the location of the regulated transcript. Results of the binomial test with (**A**) known haplotypes, (**B**) haplotypes inferred by PHASE [17] and (**C**) haplotypes inferred by fastPhase [16]; (**D**) results of the genotype-based contingency test, unaffected by rephasing; linear regression test using (**E**) rephased data as in B and (**F**) rephased data as in C.

**Figure 5:**
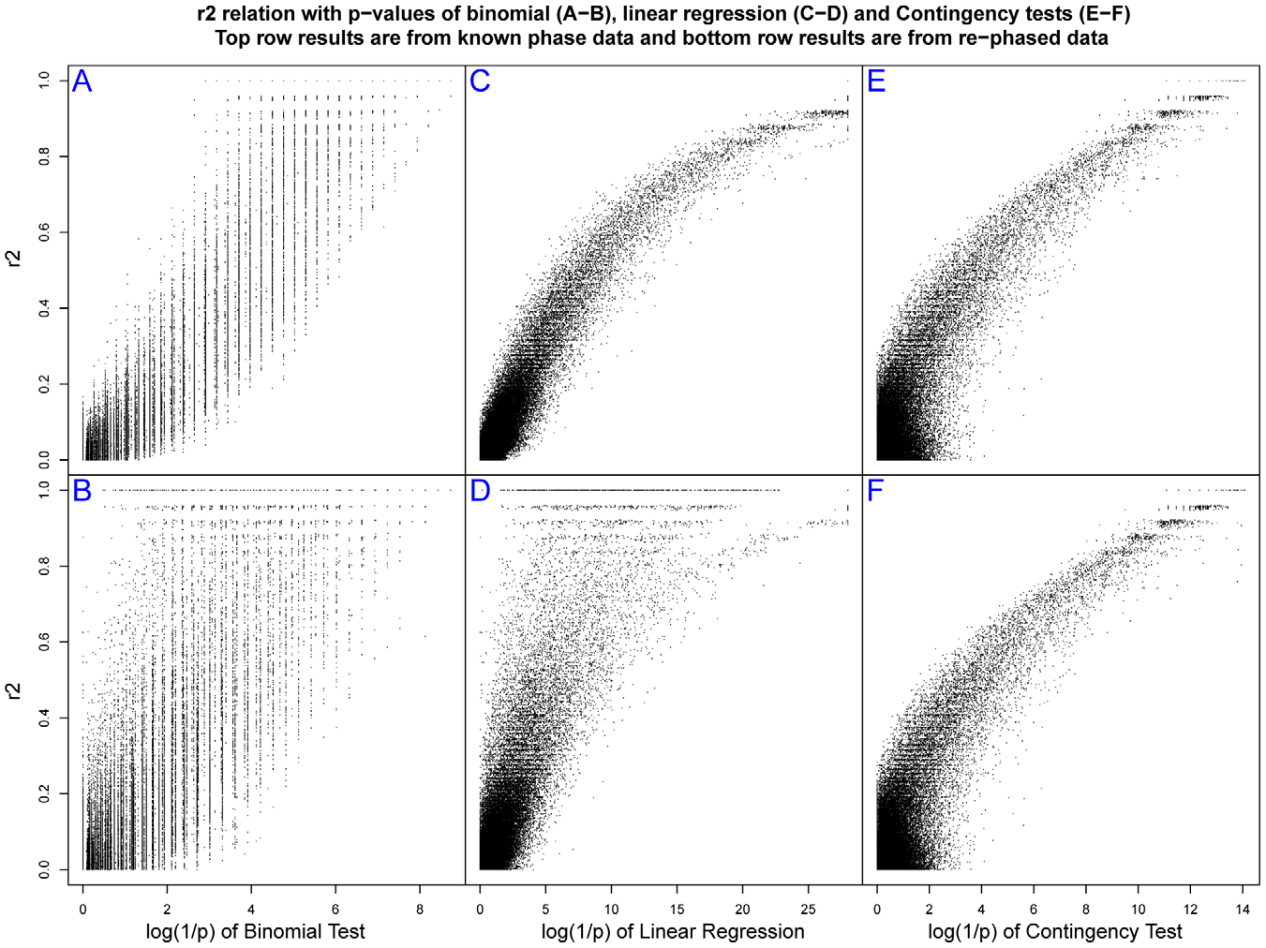
Extent of linkage disequilibrium and significance level. Plots of r^2^ coefficients between the *R* site and all tested SNPs and the corresponding log(1/p) from simulations at r frequency of ∼0.35, with known phase (upper panels) and after rephasing with PHASE [17] (lower panels), for the binomial (**A** and **B**), linear regression (**C** and **D**) and contingency tests (**E** and **F**).

**Figure 6:**
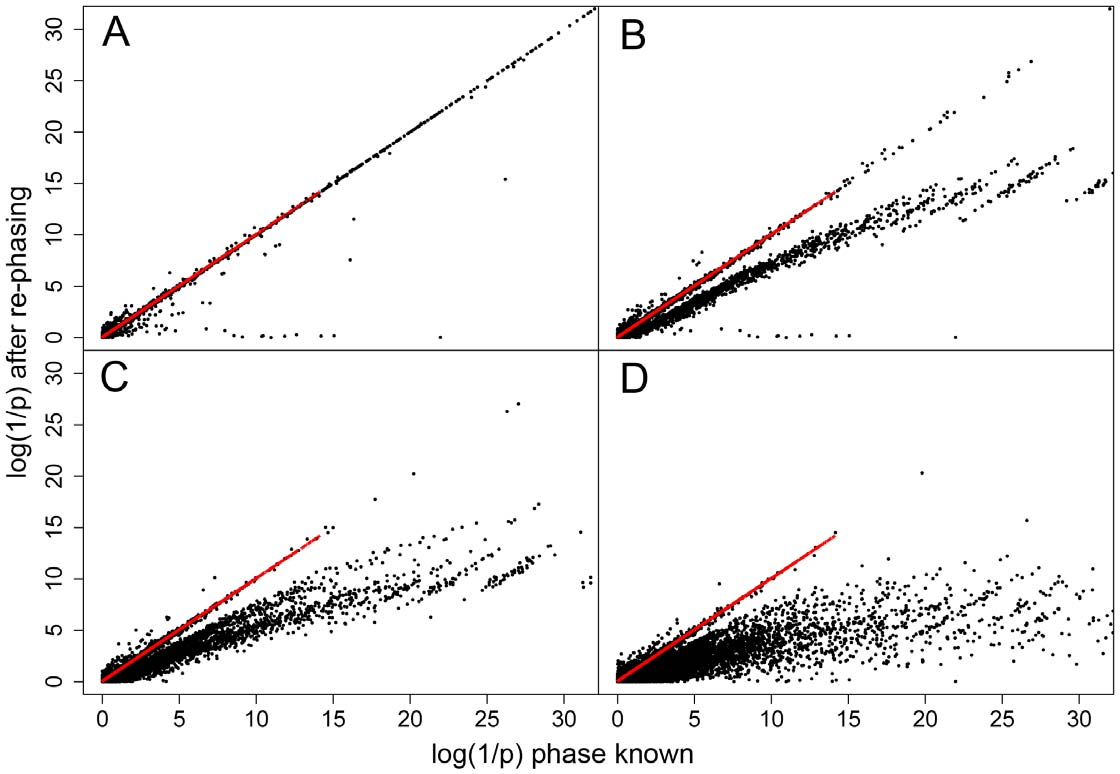
Comparison of log(1/p) values obtained before and after rephasing with PHASE. Simulations at r frequency of ∼0.35 (i.e. around 23 AI individuals out of a total of 50) were used and results were separated according to the rephasing quality evaluated as (**A**) zero, (**B**) one, (**C**) two and (**D**) three or more, AI individuals with phase switch error.

To address these issues we examined the effect of chromosomal phasing in a situation when the regulated transcript is located at a certain distance from its *R* site, separated by a recombination hotspot placed in the middle of 100 kb as illustrated in Figure 4. We selected simulations assigning a regulatory site at a given r allele frequency at the beginning of the sequence. The R and r chromosomes of each AI individual were flagged with help of a heterozygous SNP Aa at the other end within the transcribed portion of the sequence (Figure 4). After rephasing, the A and a alleles were used to define R and r chromosomes and the p values of the SNPs surrounding the original *R* site were assessed again. The presence of a single hotspot (here defining a genetic distance of ∼0.1 cM) between the virtual start site of transcription and the regulatory region was sufficient to cause a dramatic loss of power of the haplotype-based tests. Overall, for simulations at r frequency of ∼0.35, there is a loss of 98.7% (binomial) and 91.1% (linear regression) of significant SNPs (at *p*<0.01 level) after chromosome phasing using fastPhase [16] and 29.7% and 19.7%, respectively when using PHASE [17] (compare Figure 4B with C, and E with F). Therefore, from now on we will only present results obtained with better performing PHASE software. These results are shown in Figure 5 in the form of plots of the log(1/p) values of all SNPs as a function of their corresponding r^2^ coefficients with the *R* site. The upper panels illustrate how the three tests perform when phase is exactly known, and how their log(1/p) values relate to the LD coefficient r^2^. After rephasing, there is a substantial decrease of log(1/p) values in haplotype-based tests but not in the contingency test (see also Figures S1, S2, S3 for the data at other frequencies of r). Furthermore, we evaluated the performance of the tests by comparing the log(1/p) values when the extent of phasing errors was known. After rephasing, we extracted the data sets where all AI individuals were in phase, i.e. without phase switch error between the regulatory and the transcribed region. We also separated simulations where switch errors were observed in only one individual, in two individuals and in three or more individuals. In simulation experiments at r frequency of ∼0.35, the phase was conserved in all AI individuals (n = 23) in 15.5% of simulations, in 24% of the simulations we found switch error one individual, in 25% in two individuals and in three or more in the remaining 35% of simulations (Table 2). Figure 6 compares log(1/p) values obtained in these four data sets before and after rephasing using the contingency (red dots) and the linear regression test (black dots). Due to phasing errors the drop in log(1/p) values is only observed in the case of linear regression test. While already noticeable in the data sets without switch errors in AI individuals, the effect becomes dramatic when two or more individuals are affected (see also Figures S4, S5, S6). It can thus be expected that, with an increasing genetic distance between regulated and regulatory regions, the haplotype-based tests will become even more vulnerable. In other words, an accumulation of phasing errors may preclude the efficient use of haplotype based-tests in mapping regulatory regions that are located far from their regulated transcripts [3], [5], [18]

**Table 2:** Phase switch errors in AI individuals due to rephasing (%).

	r frequency			
Number of errors	0.15	0.35	0.50	0.85
0	34.6	15.5	14.6	43.5
1	33.6	24.3	20.8	27.9
2	20.3	25.4	25.0	16.0
3 or more	11.5	35.0	39.8	12.5

Phase switch errors between the direction of AI and the original haplotype phase of R and r alleles observed after rephasing (using PHASE) the simulation data for different r frequencies.

If phase is exactly known the linear regression test attains the highest log(1/p) values (>28 versus 14.08 and 9.93 for the contingency and binomial test, respectively). On the other hand, the average log(1/p) of about 3, considering all SNPs with p<10^−2^ threshold, is very similar in the three tests (Table S1). Because the spread of the log(1/p) values is test dependent, the same numerical value will have different weight in different tests. We also observe that p value is not always correlated with the proximity to the *R* site. In Figure 4E and 4F few “significant” SNPs are found separated from the regulatory region by a recombination hotspot. The proportion of such SNPs is not negligible and is the highest in the case of linear regression and the lowest in the case of the contingency test for the rephased data (Table 3). Moreover, linear regression also appears least precise in pinpointing the location of the R site, considering the relative position of the five most significant SNPs (Figure S7).

**Table 3:** Proportion of significant SNPs separated from regulatory region by a recombination hotspot (as in Figure 4).

	r frequency			
	0.15	0.35	0.50	0.85
Binomial Test				
Phase known	5.3	6.5	9.4	23.2
After re-phasing	9.6	9.0	12.8	32.4
Contingency Test				
Phase known	12.9	5.7	7.1	23.4
After re-phasing	12.9	5.7	7.1	23.4
Linear Regression				
Phase known	17.4	12.5	14.2	27.7
After re-phasing	20.8	15.2	17.2	30.9

A hotspot has been simulated between the transcript locus and the regulatory rSNP as illustrated in Figure 4.

Another issue is that of multiple-allelic (e.g. combination of the effect of two or more closely related sites) or multiple-loci regulation, whereby the same alleles of a linked polymorphic site in different individuals can be variably associated with either up- or down-regulation [19], [20]. Potentially, this could reduce the power of tests that require the measured effect of up- or down-regulation to be always associated with the same parental haplotype. For example, two independent adjacent mutations may affect a regulatory site, such that it becomes effectively tri-allelic. However, the third allele (formed by two-SNP haplotype) needs to be present at an appreciable frequency. Otherwise the two remaining alleles would dominate, making the site to behave as effectively bi-allelic. Therefore, in our simulations we assigned similar frequencies to the three alleles. We considered two genealogical positions (Figure 2) with the second derived allele to be on the background of the first (below) or to occur independently on the background of the ancestral allele (parallel). The results presented in Table 4 show that the three tests should also perform well in mapping regulatory regions more complex than the bi-allelic ones.

**Table 4:** Power and FPR in simulation experiments of a tri-allelic *R*-site.

	Power (%)		FPR (%)
Position	Parallel	Below	
Test			
Binomial	58	38	0.3
Contingency	39	37	1
Linear Regression	64	47	1

Power is separately evaluated for the two possible genealogical positions above/parallel and above/below of the derived alleles r1 and r2. The frequencies were 0.4, 0.3 and 0.3 for the ancestral haplotype (R1R2), the intermediate (R1r2 or r1R2) and the derived (r1r2), respectively. FPR was calculated by randomly assigning 33 individuals as AI positive.

### Experimental examples

We used two different datasets obtained with cell lines representing individuals of European descent from the CEPH collection. The first dataset obtained using Illumina genotyping arrays in 54 lymphoblastic cell lines by Ge et al. [6], was analyzed in the context of HapMap2 genotypes [11]. In this study ASE was considered as a continuous variable, representing the intensity of the difference of normalized expression between two chromosomes, so called AI index. To convert AI into a categorical variable, i.e. AI or non-AI, we considered the examined transcripts to be in AI when their AI index was ≥|0.1| [6]. The second dataset was obtained by second generation sequencing [7] of mRNAs from 57 lymphoblastic cell lines and by matching the sequencing results with the corresponding HapMap3 genotypes [10]. Here the difference between the observed levels and the expectation of equal allelic transcription at p<0.01 was used as indicator of AI [7] (see also Methods).

**Figure 7:**
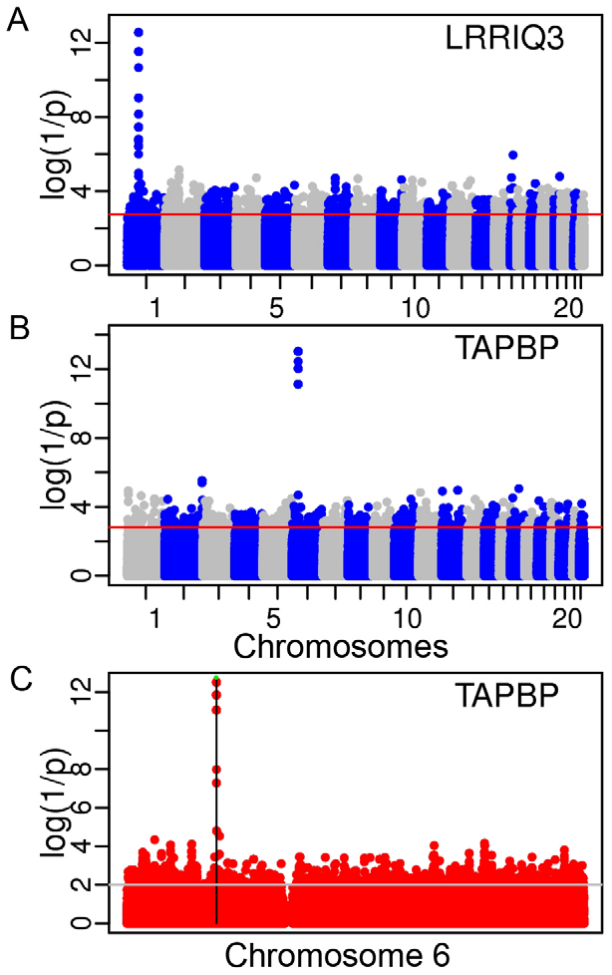
Manhattan plots of *p*-values from the contingency test. (**A**) for all autosomes using HapMap2 polymorphisms and AI data for LRRIQ3; (**B**) using HapMap3 polymorphisms and AI data for TAPBP; and (**C**) using 1000 genomes sequences for chromosome 6 and the same AI data for TAPBP.

**Figure 8:**
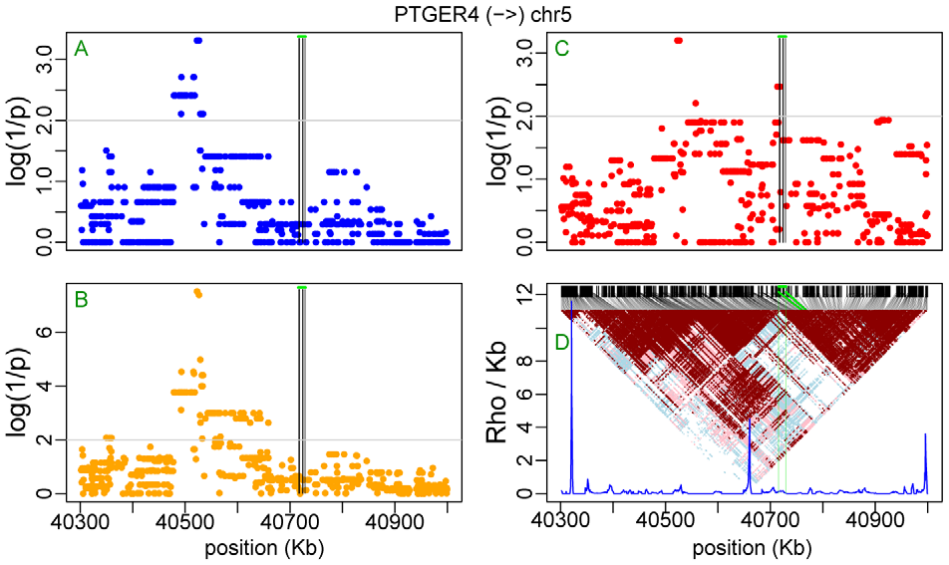
Mapping regulatory sites for PTGER4. Plots of *p*-values for HapMap2 SNPs using binomial test (**A**), linear regression test (**B**) and contingency test (**C**). Vertical black lines identify SNPs that were used as informative markers within the transcript and the green horizontal line corresponds to the analyzed transcript. The linkage disequilibrium triangle and recombination intensity profile of the population recombination rate (ρ/kb estimated by InfRec [22]) are shown in (**D**), where, black lines connect SNPs distributed according to sequence position (upper part) with their position in the LD triangle and vertical green lines delimit the size of the analyzed transcript. Arrow on the top indicates transcription direction.

**Figure 9:**
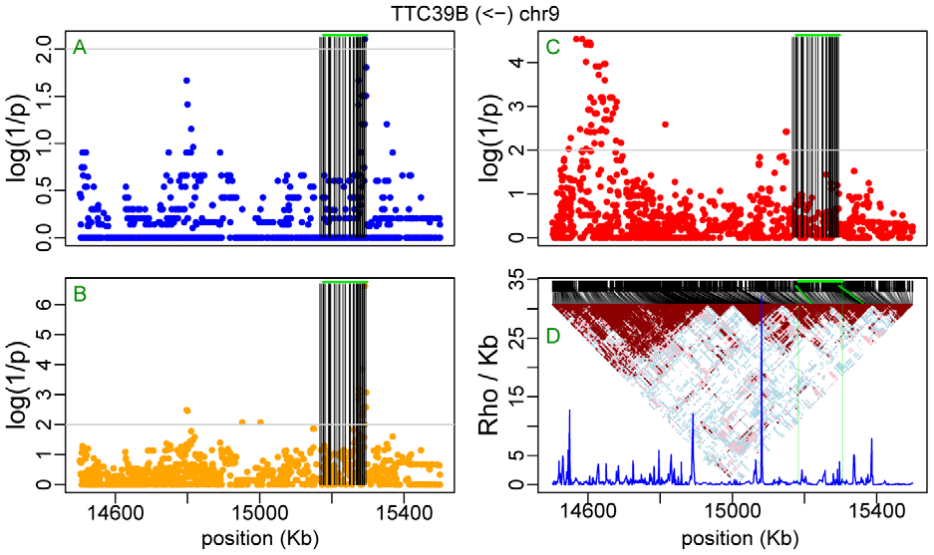
Mapping regulatory sites for TTC39b. Plots of *p*-values for HapMap2 SNPs using binomial test (**A**), linear regression test (**B**) and contingency test (**C**). Vertical black lines identify SNPs that were used as informative markers within the transcript and the green horizontal line corresponds to the analyzed transcript. The linkage disequilibrium triangle and recombination intensity profile of the population recombination rate (ρ/kb estimated by InfRec [22]) are shown in (**D**), where, black lines connect SNPs distributed according to sequence position (upper part) with their position in the LD triangle and vertical green lines delimit the size of the analyzed transcript. Arrow on the top indicates transcription direction.

Figure 7A shows the contingency test analysis of the LRRIQ3 AI data from Ge et al. [6] including SNPs from all autosomes. A similar analysis of the TAPBP transcript based on AI data from Montgomery et al. [7] is shown Figure 7B. It is repeated in Figure 7C using the full sequence information of chromosome 6 obtained from the 1000 genomes project [21]. In both loci the analysis revealed single candidate regulatory region overlapping the examined transcript (Figures S8 and S9). Note that in Figure 7A, the second minor peak on chromosome 15 is an artifact caused by coincidental concentration of unlinked singleton SNPs. As expected, the contingency test becomes especially practical when looking for regulatory sites that are far from the affected gene. A classic example is PTGER4 [6], [14] with the regulatory region located about 200 KB upstream of its transcription start site. Here, the linear regression test, which performs the best in terms of log(1/p), the binomial and the contingency test all point to four AI-associated sites (rs7720838 at map position 40522653 bp; rs7725052 at 40523027 bp; rs9283753 at 40526366 bp; rs10440635 at 40526547 bp – seen on the upper left in Figure 8A and 8B). The rs7720838 SNP, which was already reported previously (Table 5) is in complete LD with the three others. In turn, in the case of TTC39b, it is only the contingency test that highlights a potential regulatory region at about 600 KB downstream from the gene (Figure 9C; see also Table 5). The failure of the binomial and linear regression test (Figure 9A and 9B) to pinpoint the same region as a regulatory candidate is presumably related to a greater genetic distance separating it from the regulated TTC39b transcripts than in the case of PTGER4 (600 vs. 200 Kb and even greater difference in genetic distances when comparing ρ, the population recombination rate intensities in Figures 8D and 9D). Note, however, that in the same time, both haplotype based tests reveal a number of significant SNPs (one highly significant, p = 2.5×10^−7^, in the case of linear regression) among those within the transcript itself and used as informative markers for the detection of AI.

**Table 5:** Examples of sites identified in previous studies.

Transcript [direction: >; <]	rSNP	Sequence position	Reference
PTGER4>	chr 5	40715789–40729594	
	rs7720838	40522653	1
	rs7725052	40523027	
	rs9283753	40526366	
	rs10440635	40526547	
TTC39b<	chr 9	15176585–15297244	
	rs10481503	14650700	2*
	rs10481504	14650873	2*
	rs9298706	14668730	2*
LRRIQ3<	chr 1	74264290–74436459	
	rs1384883	74274065	3
	rs6676622	74282393	4*
	rs1032082	74304575	4*
	rs11210404	74310840	4*
	rs1483795	74315515	4*
	rs10789387	74332999	4*
	rs10789388	74341340	4*
	rs4142948	74344939	4*
	rs11806946	74436297	5
TAPBP<	chr 6	33379710–33389967	
	rs469064	33358454	2*
	rs455567	33360093	2*
	rs446735	33363081	2*
	rs463260	33364962	2*
	rs464865	33365164	2*
	rs3130018	33398380	2*
	rs2073525	33398803	2*
	rs3130267	33414772	2*
	rs3130270	33416199	2*
404053>	chr 9	21684732–21687392	
	rs751173	21697372	6
404105<	chr 20	29336791–29338299	
	rs2180566	29482515	7
	rs6059244	29474144	7
BAT2>	chr 6	31696429–31713533	
	rs805257	31742172	8*
	rs755714	31717792	8*
	rs2736172	31698877	9*
	rs805297	31730585	9*
FMO4>	chr 1	169550110–69577847	
	rs1963273	169589070	9*
GUCA1b<	chr 6	42259000–42270672	
	rs4714579	42282456	10
KIF16b<	chr 20	16307450–16502078	
	rs3746786	16515202	10
	rs2277777	16518934	5
	rs6075078	16519466	5
LTA>	chr 6	31648684–31649608	
	rs2844484	31644203	11,12
	rs2239704	31648120	13
MDGA1<	chr 6	37708262–37773744	
	rs6938061	37782317	14

References for Table 5: 1. [14]; 2. [7]; 3. [29]; 4. [31]; 5. [30]; 6. [32]; 7. [33]; 8. [38]; 9. [37]; 10. [6]; 11. [36]; 12. [34]; 13. [35]; 14. [45]. *: Reference found through the eQTL browser (http://eqtl.uchicago.edu/cgi-bin/gbrowse/eqtl/).

While in many instances the contingency test outperforms the binomial one (Figures S8, S9, S10, S11, S12, S13), they often perform equally well (Figures S14, S15, S16) or the binomial one appears more efficient (Figure S17). As a rule log(1/p) values are much higher in the case of linear regression. Both experimental datasets [6], [7] provided high-quality data to reveal the presence of ASE (Figure S18), although the significant sites did not always fully overlap due to differences in the SNP catalogs between HapMap2 and HapMap3. Table 5 lists selected SNPs identified by us and known previously from other studies to provide additional support and verification of our approach. SNPs density can be easily increased by incorporating into the analysis the sequence data of the 1000 genomes project available for the same population samples (Figure 7C).

### Empirical False Positive Rate

In power calculations based on the empirical data we examined all HapMap2 autosomal SNPs (>2.5 million) in the set of 54 individuals from Ge et al. [6], assuming the presence of AI in a range of randomly chosen individuals (Table 6). These estimates show that the overall FPR is less than 1% for all tests at these conditions. We can therefore presume that scanning the whole genome will rarely give rise to misleading regions of significant SNPs. Especially considering most false positives would occur alone, while significant SNPs are expected to occur in clusters representing genomic segments in LD. To identify such segments we additionally examined [22] the recombination rate and LD profiles in the identified regions, as shown in Figures 8C and 9C.

**Table 6:** Empirical False Positive Rate estimates.

	FPR (%)				
AI individuals	5	10	15	20	29
Test					
Binomial	0.00	0.01	0.1	0.17	0.26
Contingency	0.60	0.66	0.69	0.72	0.72
Linear Regression	0.06	0.34	0.34	0.54	0.59

Percent of SNPs showing *p*-values below 0.01, after randomly assigning AI to 5, 10, 15, 20 and 29 individuals out of the 54 considered (based on the set of Ge et al.). Based on 20 whole genome scans for 10, 15 and 20 AI individuals and on 20 scans of chromosomes 1 to 4 for 5 and 29 AI individuals.

## Discussion

Variation in transcriptional regulation of gene expression plays a significant role in determining the diversity of human phenotypes. The components of transcriptional control include *cis*-acting regulatory elements that may act across large genomic distances, hundreds or thousands of Kb away from the transcript they regulate [18]. Studies of ASE indicate that allele-specific differences among transcripts within an individual can affect up to 30% of loci and, at the population level, ∼30% of expressed genes show evidence of *cis* regulation by common variants [6]. In population studies, an even larger proportion of genes showed ASE that could not be mapped, which could be ascribed to rare genetic variants or epigenetic effects [8]. However, it is also possible that some distal regulatory regions have escaped detection because they are located far from the regulated transcript. First, because of large distance they could have been left unexplored, and second, because the mapping could have failed, if tests required knowledge of the chromosomal phase. Sample size, the reliability of genotyping and the accuracy and completeness of AI ascertainment, will affect the outcome of all tests. Because genotype-based test is independent of chromosomal phasing and phasing errors, its mapping efficacy is also unaffected by genetic distance separating regulatory site from the regulated transcript. Phasing errors are unavoidable, even when using best haplotype-phase inference algorithms [23]. Their number increases with increasing chromosomal distances and with the number of recombination hotspots in between. They may be also more frequent in admixed individuals and in newly studied populations with unknown haplotype catalogs [24]. The most accurate algorithms, such as PHASE require very long computation times (on a regular 2 GHz computer), which may extend from days to months for sets of hundreds of thousands of SNPs in a hundred genotyped individuals [16], [25]. While this was not an issue with our simulated data sets of 50 diploid individuals and an average of about 500 SNPs (θ = 100), it still took more than 50 min on 2.67 GHZ processor. Faster programs exist and, for example, it takes about 2 min run to phase the same data set using ShapeIT [25]. Regrettably, the speed is reached at the expense of accuracy which varies as a function of the sample size and the amount of markers [26]. In other words, using genotype test is less computationally demanding, we gain in time and in accuracy when phasing errors are an issue.

Our analyses on real data were carried out in very well phased CEU individuals from the HapMap project, where phasing was additionally improved by using child-parental trios. In most cases the haplotype-based binomial test worked equally well or even better than the genotype-based test as judged by significance levels. However, while both tests “found” the PTGER4 *cis*-regulatory segment located almost 200 kb upstream of this gene (Figure 8), our binomial and linear regression test failed to identify such a region more than 500 kb downstream of the TTC39b transcript (Figure 9). When we rephased the genotype data from Figure 8 using fastPhase [16] (but not PHASE [17] or Shape-IT [25]), the upstream regulatory segment of the PTGER4 transcript also became “invisible” in the haplotype based tests. Therefore, when chromosomes are well phased these tests can be expected to lead to the same or similar overlapping results (e.g. Figure 8). Importantly, these two examples (Figures 8 and 9) illustrate well the problem of locating regulatory variants/regions from ASE data. An informative marker whose alleles are at least partly consistent with the direction of up and down transcription control may be revealed as significant. The chances that this happens are increased when many such markers are used (or when many transcripts are tested with highly informative markers) and haplotype-based tests seem to be more vulnerable to this kind of error. Lack of statistical significance in the genotype-based vs. haplotype-based tests of a number of SNPs representing the informative markers zone, as in Figure 9, strongly suggests that these do not indicate the location of the regulatory region but rather reflect a partial overlapping in heterozygosity and phase between marker and regulatory sites. In the reverse case, lack of statistical significance in the haplotype-based vs. the genotype-based test may also suggest a different genetic mechanism. For example, in the case of an imprinted locus, when one of the parental chromosomes is silenced, a signal of AI will be observed [27]. This is observed in the SNRPN locus (Figure S19) reported to be imprinted [28], and in the L3MBTL locus (Figure S20) where haplotype based analyses failed and the contingency test revealed as significant the informative markers and other SNPs in their linkage group. Likewise, an “artificial” AI signal could also reflect random mono-allelic expression in a fraction of individuals (cell lines), due to aberrant methylation of the genome [27]. In other words, combining the results of haplotype and genotype-based tests may provide leads to AI-causing mechanisms other than due to genetic variation within regulatory elements. In Table 5 we listed selected SNPs found by us, which were earlier reported in either different GWAS or expression studies by others. For example, PTGER4 rs7720838 was found associated with the risk of Crohn's disease [14]. The rs1384883 SNP from LRRIQ3 was reported in a GWAS of blood pressure and hypertension [29], while other SNPs associated with the ASE of the LRRIQ3 transcript were reported in gene expression studies [30], [31]. The SNP rs751173 (transcript 404053) was reported in a study of susceptibility to cutaneous nevi [32], these associated with AI of the transcript 404105 were highlighted in a GWAS on late-onset Alzheimer disease, with rs2180566 found in the promoter of DEFB123 [33]. In turn, LTA locus with rs2844484 and rs2239704 was found in studies of cancer susceptibility and the risk of ischemic stroke [34]–[36]. All the remaining sites were earlier identified in studies of gene expression in the context of eQTL mapping. Thus our findings here can be considered as confirmatory. Interestingly, however, the three SNPs listed in the context of TTC39b, and found in the larger cluster of significant sites based on the data shown in Figure 9, were reported in the context of the PSIP1 transcript, about 150 kb upstream from TTC39b [7]. Likewise, rs1963273 identified in the context of the FMO1 transcript [37] was found here to be linked to AI within FMO4 (Figure S10) and SNPs listed for BAT2 were previously reported in LD with CSNK2B transcription [37], [38]. Do these results represent examples of synchronized transcription control, as could be suspected in the case of a gene cluster involving FMO1 and FMO4, or are they due to experimental artefacts partly caused by phasing errors?

In contrast to other analyses, which may consider the intensity of the ASE signal [6], the tests introduced here are based on a categorical classification of the individuals studied as AI or non-AI. The mapping of regulatory variation thus critically depends on the quality of AI measurement as well as on the number of intergenic informative marker SNPs serving to ascertain AI status of the sampled individuals. Detecting or confirming the presence of AI is not the same as mapping regulatory variants. For the first it is sufficient to demonstrate that two parental copies of a gene are differentially expressed. Mapping requires sufficiently large samples where ideally all AI expressing individuals can be detected. Power and FPR of the mapping tests depend upon the characteristics of the polymorphic sites in LD within a regulatory segment (Table 1). These characteristics, which include their allelic frequencies, genealogical positions and r^2^ relative to the *R* site, change with the increasing r frequency (Figure S21 and Table S2). Selecting simulations for the presence of a derived allele above certain frequency level eliminates a portion of coalescence trees representing particular genealogical histories that cannot “accommodate” sites with a derived allele above certain frequency level. While among 2000 simulated genealogies all “carry” a site with a derived allele frequency of ∼0.15, only 897 (45%) genealogies carried sites with a derived allele frequency of ∼0.85 (Table S2). This leads to a progressive distortion (as compared to neutral expectation) of allelic frequency spectra of SNPs in LD with the *R* site at increasing frequency of its r allele (Figure S22), which affects the proportions of significant SNPs in each position category between the tests. Knowing the number of AI individuals we may estimate heterozygosity and thus relative R and r allele frequencies. The knowledge of the expected genealogical positions of SNPs that are tightly linked with the *R* site allows us to better understand differences between outcomes of different tests (Table S2). When combined with the analysis of the regulatory region haplotypes it may be also useful in finding the regulatory site itself.

Systematic use of genotype-based tests in concert with haplotype-based tests may be the most advisable mapping strategy. Unfortunately, haplotype-based tests will always suffer from phasing uncertainty inherent to the data itself, especially when the number of samples precludes the use of computationally slow but more reliable phasing algorithms. Using the genotype-based test, the phasing step can be simply postponed saving time and related costs. The best outcomes can be expected with high quality data maximizing ascertainment of AI individuals. The utility of the genotype-based test will increase with the application of new sequencing methods that improve transcript quantification thus providing more reliable assessment of AI status. Without phasing uncertainty, the genotype-based test should pave the road to the identification of *cis*-regulatory variants that could have escaped detection due to their distal location [4], [18]. Finally, the current trend of functional genomics based on next-generation sequencing makes it possible to interrogate allelic functional effects beyond transcription [8]. Any inheritable phenotypes that can be categorized such as AI here, identifying the underlying heterozygotes, can be mapped using presented protocols. The genotype test can also be extended to compare phenotyped individuals that may represent different genotype combinations. Our approaches can be generalized to map for causes of differential DNA-protein interactions or active chromatin, both shown to be inheritable in recent studies [39], [40].

## Methods

### Evaluation of statistical tests by simulation experiments

Coalescent simulations were performed using the programs ms [41] and msHot [42]. A typical experiment consisted of 2000 simulations of 50 individuals (i.e., 100 sequences) with a population mutation rate Θ of 50. Considering effective population size *Ne* of 12 500 individuals and mutation rate *μ* of 2×10^−8^ per nucleotide per generation leads to the sequence of 50 Kb. In the presence of recombination, the population recombination rate ρ was set to 25. When hotspots were simulated, 90% of all recombinations occurred within a single hotspot of 1 kb in the middle of the sequence. Genotypes were constructed by randomly pairing simulated haplotypes and the resulting Rr heterozygotes were considered as AI individuals.

In each simulation, among the entire set of simulated SNPs, we selected an rSNP with a specific frequency at its derived allelic state, r frequency of 0.15, 0.35, 0.5 and 0.85 (or in the closest interval) and assigned it as an *R* site. The remaining mutations were considered as accompanying SNPs (*A* sites). In practice, our r-alleles chosen from simulation experiments had frequencies of 0.144±0.021, 0.337±0.046, 0.495±0.056 and 0.847±0.060, respectively. Importantly, not all of the simulations carried derived alleles above 0.15, such that from 2000 simulations in the absence of recombination, only 1954 remained in a sample with r-allele frequency of 0.35, 1681 with r frequency of 0.5 and 897 with r frequency of 0.85. The data obtained in each simulation experiment were used to estimate power and FPR of the three tests. To evaluate the extent of linkage disequilibrium between rSNP and other sites we used r^2^ coefficient [43].

Power was estimated as the fraction of significant sites (p<0.01) over all sites or over all sites with MAF of 5% or more. To evaluate FPR (type I error), we first calculated the mean number of Rr heterozygotes corresponding to AI individuals in each experiment and then, we randomly assigned AI status to the same number of simulated individuals. The number of significant sites over all SNPs, or those at MAF≥5%, yields the FPR. The number of simulations having at least one significant SNP was also computed for the FPR.

### Phasing errors and incomplete ascertainment of AI individuals

To test the effect of phasing haplotypes from the genotypes we compared the results obtained from the diploid individuals created using original simulated haplotypes with the ones using haplotypes inferred from the reconstructed genotypes by fastPhase [16] and PHASE [17]. In addition, we carried simulations as described above for 50 individuals, except that there were 1000 simulations, Θ and ρ were set to 100 and 50, respectively. Thus sequences were 100 kb long, a recombination hotspot 1 kb wide was placed in the middle of the sequence with 90% percent of all recombinations occurring within the hotspot. We defined AI individuals as heterozygous for the *R* site preselected for desired r frequency and located at one end of the sequence. In AI individuals, we used heterozygous SNPs at the other end of the sequence to keep track of the phase of AI after rephasing (see Figure 4). This way the effect of phasing errors between a putative *R* site and the regulated transcript separated by hotspots can be evaluated.

### Tri-allelic R site

To estimate the performance of the tests given the tri-allelic *R*-site, we used simulations under the same conditions with no recombination involved. We combined two mutations to obtain three haplotypes that would confer distinct levels of allelic expression. Let us denote R1 and r1 the ancestral and derived alleles at the first site, and R2 and r2 the corresponding alleles at the second site. We arbitrarily assumed the lowest expression level to be associated with the ancestral haplotype R1R2. The first mutation that leads to the haplotypes r1R2 or R1r2 will be associated with an intermediate expression and the second mutation leading to the r1r2 variant was assigned to confer the highest expression level. In this three-allelic model, we considered frequencies of 0.4, 0.3 and 0.3 for the ancestral, intermediate and the derived variant, respectively.

### Empirical evaluation of statistical tests

To evaluate the distribution of the observed *p*-values, we used the same 54 individuals with their HapMap2 genotypes that were analyzed by Ge et al [6]. From this sample, we randomly chose 5, 10, 15, 20, 25 or 29 individuals, as if they were in AI. Subsequently, we evaluated *p*-values for each SNP along the whole genome (for 5 and 29 individuals rather than whole genome we only used chromosomes 1, 2, 3 and 4 instead). This was repeated 20 times for each number of randomly assigned AI individuals.

### Using the data

The data on differential ASE determined in 54 lymphoblastic cell lines are from Ge et al. [6]. Briefly, several markers were used along the genome to evaluate allelic expression. Markers are considered informative when they are heterozygous and their expression intensity is sufficiently high as in R = log(X_raw_+Y_raw_)>1000. The AI indices measured by |Δ _het ratio_| evaluate the difference in expression level between two allelic chromosomes and we set the threshold over which it indicates differential ASE to 0.1. However, the results were not always unequivocal, i.e., with all informative markers reporting consistent results. In practice, there is a substantial variance in signal intensity and in AI indices between informative marker-SNPs within a single individual [44]. Since our tests require partitioning AI and non-AI individuals as well as possible, we carefully evaluated AI for each individual. We used the mean AI either from all informative markers (heterozygote markers within that individual) or considering only significant markers (R>1000). In both cases, the individual was considered in AI when his AI index was over 0.1.

We also used second generation sequencing results from Montgomery et al. [7] where differential allelic expression was examined by counting transcripts in heterozygous individuals, using polymorphic markers from the HapMap3 project in the 113 HapMap lymphoblastic cell lines representing individuals of European descent. The presence of differential allelic expression was assessed based on a binomial probability of differences in raw counts for each allele with correction for reference mapping biases. When an individual had at least one marker with a *p*-value<0.01, he was considered in AI.

The extent of genetic distance between SNPs of the analyzed DNA regions was assessed by LD-triangles, representing the significance of association between SNPs based on χ^2^ or Fisher's exact test, and by plotting the intensity profiles of the population recombination rate ρ obtained by InfRec [22].

## Supporting Information

Figure S1 **Extent of linkage disequilibrium and significance level.** Plots of r^2^ coefficient between the *R* site and all tested SNPs and the corresponding log(1/p) from simulations at r frequency of ∼0.15, with known phase (upper panels) and after rephasing with PHASE (lower panels), for the binomial (**A** and **B**), linear regression (**C** and **D**) and contingency test (**E** and **F**).
(TIF)

Figure S2 **Extent of linkage disequilibrium and significance level.** Plots of r^2^ coefficient between the *R* site and all tested SNPs and the corresponding log(1/p) from simulations at r frequency of ∼0.5, with known phase (upper panels) and after rephasing with PHASE (lower panels), for the binomial (**A** and **B**), linear regression (**C** and **D**) and contingency test (**E** and **F**).
(TIF)

Figure S3 **Extent of linkage disequilibrium and significance level.** Plots of r^2^ coefficient between the *R* site and all tested SNPs and the corresponding log(1/p) from simulations at r frequency of ∼0.85, with known phase (upper panels) and after rephasing with PHASE (lower panels), for the binomial (**A** and **B**), linear regression (**C** and **D**) and contingency test (**E** and **F**).
(TIF)

Figure S4 **Comparison of log(1/p) values obtained before and after rephasing with PHASE.** Simulations at r frequency of ∼0.15 (i.e. around 23 AI individuals out of a total of 50) were used and results were separated according to the rephasing quality evaluated as (**A**) zero, (**B**) one, (**C**) two and (**D**) three or more, AI individuals with phase inversion.
(TIF)

Figure S5 **Comparison of log(1/p) values obtained before and after rephasing with PHASE.** Simulations at r frequency of ∼0.5 (i.e. around 23 AI individuals out of a total of 50) were used and results were separated according to the rephasing quality evaluated as (**A**) zero, (**B**) one, (**C**) two and (**D**) three or more, AI individuals with phase inversion.
(TIF)

Figure S6 **Comparison of log(1/p) values obtained before and after rephasing with PHASE.** Simulations at r frequency of ∼0.85 (i.e. around 23 AI individuals out of a total of 50) were used and results were separated according to the rephasing quality evaluated as (**A**) zero, (**B**) one, (**C**) two and (**D**) three or more, AI individuals with phase inversion.
(TIF)

Figure S7 **Distributions of the mean distances of the 5 lowest** ***p*** **-values** ***A*** **-sites to the regulatory SNP.** Simulation results with recombination are shown by the red line (uniform) and green bars (single recombination hotspot). Those in the absence of recombination are shown by the blue line. The results from the binomial, contingency and linear regression tests are presented in downward order.
(TIF)

Figure S8 **Mapping regulatory sites for LRRIQ3 (Ge, Pokholok et al. 2009).** Plots of *p*-values for HapMap2 SNPs using binomial test (**A**), linear regression test (**B**) and contingency test (**C**). Vertical black lines identify SNPs that were used as informative markers within the transcript and the green horizontal line corresponds to the analyzed transcript. (**D**)The linkage disequilibrium triangle and recombination intensity profile of the population recombination rate (ρ/kb estimated by InfRec), where, black lines connect SNPs distributed according to sequence position (upper part) with their position in the LD triangle and vertical green lines delimit the size of the analyzed transcript. Arrow on the top indicates transcription direction.
(TIF)

Figure S9 **Mapping regulatory sites for TAPBP (Montgomery, Sammeth et al. 2010).** Plots of *p*-values for HapMap3 SNPs using binomial test (**A**), linear regression test (**B**) and contingency test (**C**). Vertical black lines identify SNPs that were used as informative markers within the transcript and the green horizontal line corresponds to the analyzed transcript. (**D**) The linkage disequilibrium triangle and recombination intensity profile of the population recombination rate (ρ/kb estimated by InfRec), where, black lines connect SNPs distributed according to sequence position (upper part) with their position in the LD triangle and vertical green lines delimit the size of the analyzed transcript. Arrow on the top indicates transcription direction.
(TIF)

Figure S10 **Mapping regulatory sites for FMO4 (Montgomery, Sammeth et al. 2010).** Plots of *p*-values for HapMap3 SNPs using binomial test (**A**), linear regression test (**B**) and contingency test (**C**). Vertical black lines identify SNPs that were used as informative markers within the transcript and the green horizontal line corresponds to the analyzed transcript. (**D**) The linkage disequilibrium triangle and recombination intensity profile of the population recombination rate (ρ/kb estimated by InfRec), where, black lines connect SNPs distributed according to sequence position (upper part) with their position in the LD triangle and vertical green lines delimit the size of the analyzed transcript. Arrow on the top indicates transcription direction.
(TIF)

Figure S11 **Mapping regulatory sites for LTA (Montgomery, Sammeth et al. 2010).** Plots of *p*-values for HapMap3 SNPs using binomial test (**A**), linear regression test (**B**) and contingency test (**C**). Vertical black lines identify SNPs that were used as informative markers within the transcript and the green horizontal line corresponds to the analyzed transcript. (**D**) The linkage disequilibrium triangle and recombination intensity profile of the population recombination rate (ρ/kb estimated by InfRec), where, black lines connect SNPs distributed according to sequence position (upper part) with their position in the LD triangle and vertical green lines delimit the size of the analyzed transcript. Arrow on the top indicates transcription direction.
(TIF)

Figure S12 **Mapping regulatory sites for GUCA (Ge, Pokholok et al. 2009).** Plots of *p*-values for HapMap2 SNPs using binomial test (**A**), linear regression test (**B**) and contingency test (**C**). Vertical black lines identify SNPs that were used as informative markers within the transcript and the green horizontal line corresponds to the analyzed transcript. (**D**)The linkage disequilibrium triangle and recombination intensity profile of the population recombination rate (ρ/kb estimated by InfRec), where, black lines connect SNPs distributed according to sequence position (upper part) with their position in the LD triangle and vertical green lines delimit the size of the analyzed transcript. Arrow on the top indicates transcription direction.
(TIF)

Figure S13 **Mapping regulatory sites for BAT2 (Ge, Pokholok et al. 2009).** Plots of *p*-values for HapMap2 SNPs using binomial test (**A**), linear regression test (**B**) and contingency test (**C**). Vertical black lines identify SNPs that were used as informative markers within the transcript and the green horizontal line corresponds to the analyzed transcript. (**D**)The linkage disequilibrium triangle and recombination intensity profile of the population recombination rate (ρ/kb estimated by InfRec), where, black lines connect SNPs distributed according to sequence position (upper part) with their position in the LD triangle and vertical green lines delimit the size of the analyzed transcript. Arrow on the top indicates transcription direction.
(TIF)

Figure S14 **Mapping regulatory sites for transcript 404053 (Montgomery, Sammeth et al. 2010).** Plots of *p*-values for HapMap3 SNPs using binomial test (**A**), linear regression test (**B**) and contingency test (**C**). Vertical black lines identify SNPs that were used as informative markers within the transcript and the green horizontal line corresponds to the analyzed transcript. (**D**) The linkage disequilibrium triangle and recombination intensity profile of the population recombination rate (ρ/kb estimated by InfRec), where, black lines connect SNPs distributed according to sequence position (upper part) with their position in the LD triangle and vertical green lines delimit the size of the analyzed transcript. Arrow on the top indicates transcription direction.
(TIF)

Figure S15 **Mapping regulatory sites for transcript 404105 (Montgomery, Sammeth et al. 2010).** Plots of *p*-values for HapMap3 SNPs using binomial test (**A**), linear regression test (**B**) and contingency test (**C**). Vertical black lines identify SNPs that were used as informative markers within the transcript and the green horizontal line corresponds to the analyzed transcript. Arrow on the top indicates transcription direction. Two crossed sites represent SNPs that were identified in the GWAS on late-onset Alzheimer disease: rs2180566 in the DEFB123 (orange line) promoter and rs6059244 more to the left (see Table 3). (**D**) The linkage disequilibrium triangle and recombination intensity profile of the population recombination rate (ρ/kb estimated by InfRec), where, black lines connect SNPs distributed according to sequence position (upper part) with their position in the LD triangle and vertical green lines delimit the size of the analyzed transcript.
(TIF)

Figure S16 **Mapping regulatory sites for MDGA1 (Ge, Pokholok et al. 2009).** Plots of *p*-values for HapMap2 SNPs using binomial test (**A**), linear regression test (**B**) and contingency test (**C**). Vertical black lines identify SNPs that were used as informative markers within the transcript and the green horizontal line corresponds to the analyzed transcript. (**D**)The linkage disequilibrium triangle and recombination intensity profile of the population recombination rate (ρ/kb estimated by InfRec), where, black lines connect SNPs distributed according to sequence position (upper part) with their position in the LD triangle and vertical green lines delimit the size of the analyzed transcript. Arrow on the top indicates transcription direction.
(TIF)

Figure S17 **Mapping regulatory sites for KIF16b (Ge, Pokholok et al. 2009).** Plots of *p*-values for HapMap2 SNPs using binomial test (**A**), linear regression test (**B**) and contingency test (**C**). Vertical black lines identify SNPs that were used as informative markers within the transcript and the green horizontal line corresponds to the analyzed transcript. (**D**)The linkage disequilibrium triangle and recombination intensity profile of the population recombination rate (ρ/kb estimated by InfRec), where, black lines connect SNPs distributed according to sequence position (upper part) with their position in the LD triangle and vertical green lines delimit the size of the analyzed transcript. Arrow on the top indicates transcription direction.
(TIFF)

Figure S18 **Comparison of the results from Ge, Pokholok et al. 2009 (left, based on HapMap2) with those from Montgomery, Sammeth et al. 2010 (right, based on HapMap3).** Plots of *p*-values for HapMap2 (left) and HapMap3 (right) SNPs using binomial test (**A**), linear regression test (**B**) and contingency test (**C**). Vertical black lines identify SNPs that were used as informative markers within the transcript and the green horizontal line corresponds to the analyzed transcript. (**D**)The linkage disequilibrium triangle and recombination intensity profile of the population recombination rate (ρ/kb estimated by InfRec), where, black lines connect SNPs distributed according to sequence position (upper part) with their position in the LD triangle and vertical green lines delimit the size of the analyzed transcript. Arrow on the top indicates transcription direction.
(TIFF)

Figure S19 **Analysis of ASE in SNRPN.** Plots of *p*-values for HapMap3 SNPs using binomial test (**A**), linear regression test (**B**) and contingency test (**C**). Vertical black lines identify SNPs that were used as informative markers within the transcript and the green horizontal line corresponds to the analyzed transcript. (**D**) The linkage disequilibrium triangle and recombination intensity profile of the population recombination rate (ρ/kb estimated by InfRec), where, black lines connect SNPs distributed according to sequence position (upper part) with their position in the LD triangle and vertical green lines delimit the size of the analyzed transcript. Arrow on the top indicates transcription direction.
(TIF)

Figure S20 **Analysis of ASE in L3MBTL.** Plots of *p*-values for HapMap2 SNPs using binomial test (**A**), linear regression test (**B**) and contingency test (**C**). Vertical black lines identify SNPs that were used as informative markers within the transcript and the green horizontal line corresponds to the analyzed transcript. (**D**)The linkage disequilibrium triangle and recombination intensity profile of the population recombination rate (ρ/kb estimated by InfRec), where, black lines connect SNPs distributed according to sequence position (upper part) with their position in the LD triangle and vertical green lines delimit the size of the analyzed transcript. Arrow on the top indicates transcription direction.
(TIF)

Figure S21 **Relation between the linkage disequilibrium between the** ***R*** **site and all tested SNPs (r^2^ coefficient), and the corresponding Minor Allele Frequency (MAF).** Green dash are for the SNPs above the rSNP, red slash are for below and blue back-slash are for parallel SNPs. The four different rSNP frequencies tested are shown.
(TIF)

Figure S22 **Folded and unfolded allelic frequency spectra from the different r frequencies sets of simulations.** Both spectra are from four subsets of 2000 simulations where a rSNP of frequency 0.15 (black), 0.35 (red), 0.5 (blue) and 0.85 (green), could be assigned. The subsets are 2000, 1954, 1681 and 897 for 0.15, 0.35, 0.5 and 0.85, respectively.
(TIF)

Table S1 **Lowest and mean p-values, expressed in log(1/p), of the significant results of the three tests performed on three sets of simulations, the “no-recombination” set, the “recombination with phase known” and the “recombination with re-phased data”.**
(DOCX)

Table S2 **Distribution of segregating sites across genealogical trees preselected to carry r alleles above certain frequency threshold and its effect on r^2^, derived allele frequency (DAF) and power of the tests in each site position category.**
(DOCX)
