# Supplementary figures and images for: Genotype-Based Test in Mapping Cis-Regulatory Variants from Allele-Specific Expression Data
Source: PLoS One. 2012 Jun 7;7(6):e38667. doi: 10.1371/journal.pone.0038667 (PMC3369843; doi:10.1371/journal.pone.0038667)

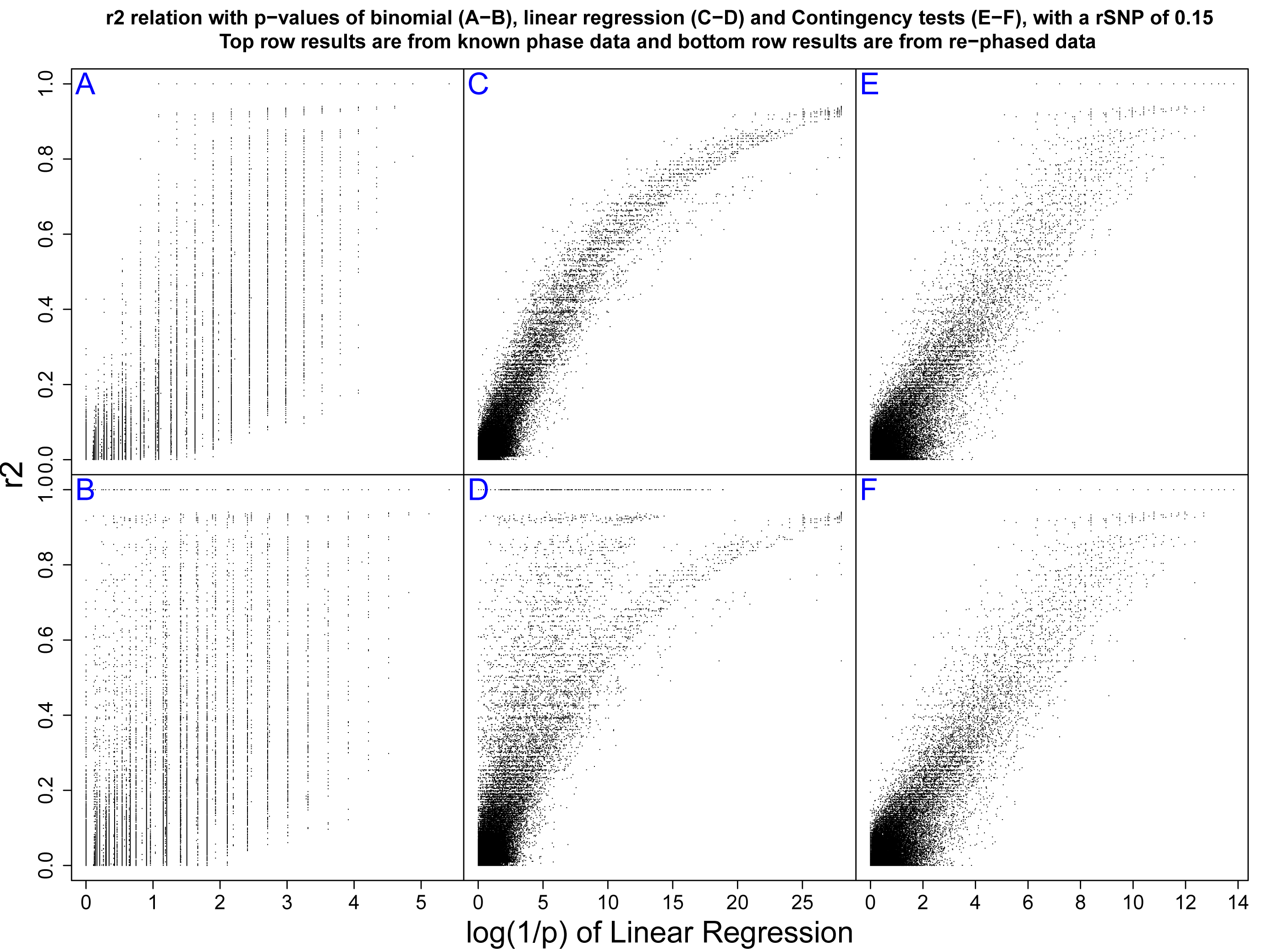

Supplement: Figure S1 — Extent of linkage disequilibrium and significance level. Plots of r2 coefficient between the R site and all tested SNPs and the corresponding log(1/p) from simulations at r frequency of ∼0.15, with known phase (upper panels) and after rephasing with PHASE (lower panels), for the binomial (A and B), linear regression (C and D) and contingency test (E and F). (TIF) [file pone.0038667.s001.tif]

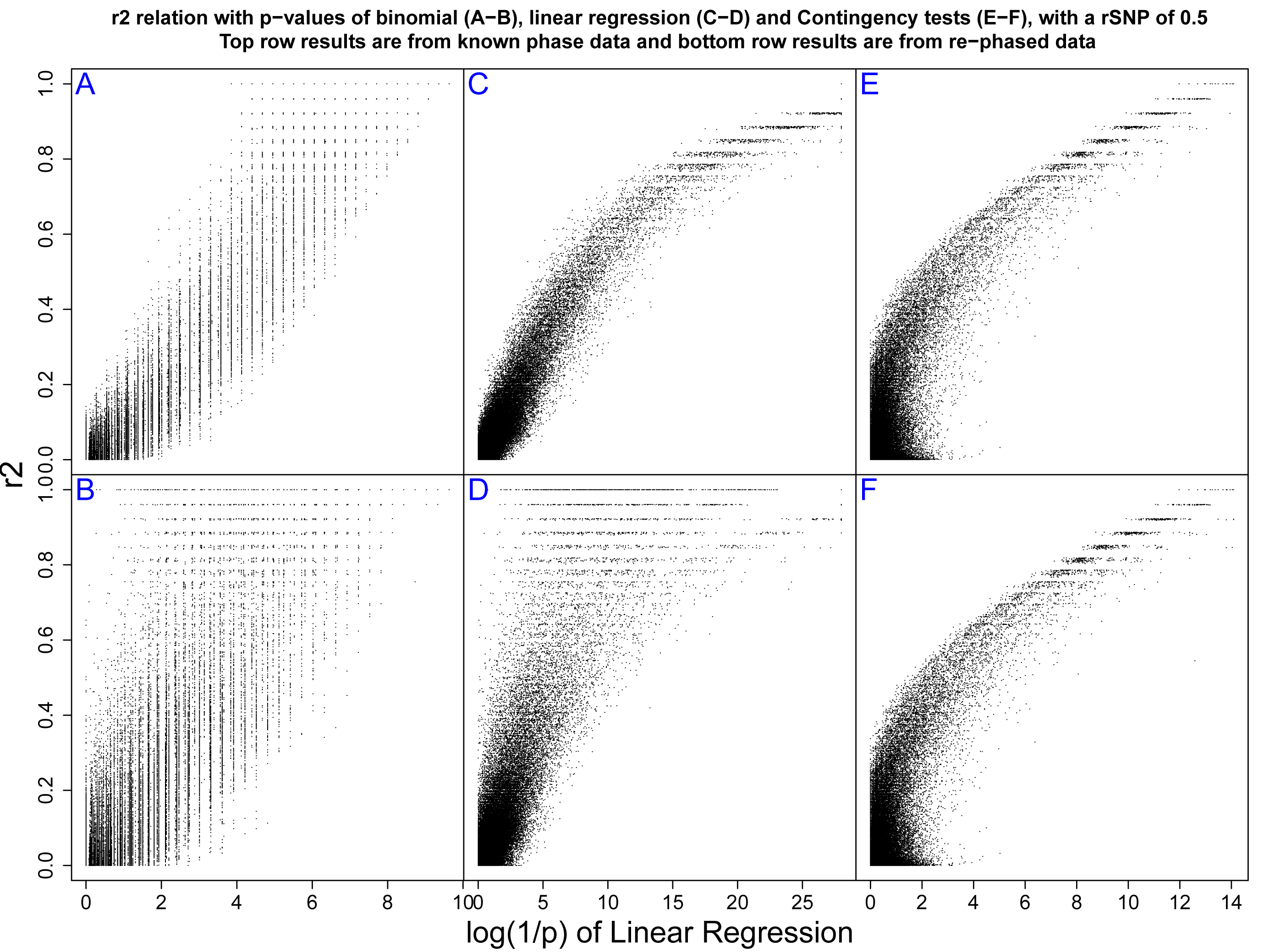

Supplement: Figure S2 — Extent of linkage disequilibrium and significance level. Plots of r2 coefficient between the R site and all tested SNPs and the corresponding log(1/p) from simulations at r frequency of ∼0.5, with known phase (upper panels) and after rephasing with PHASE (lower panels), for the binomial (A and B), linear regression (C and D) and contingency test (E and F). (TIF) [file pone.0038667.s002.tif]

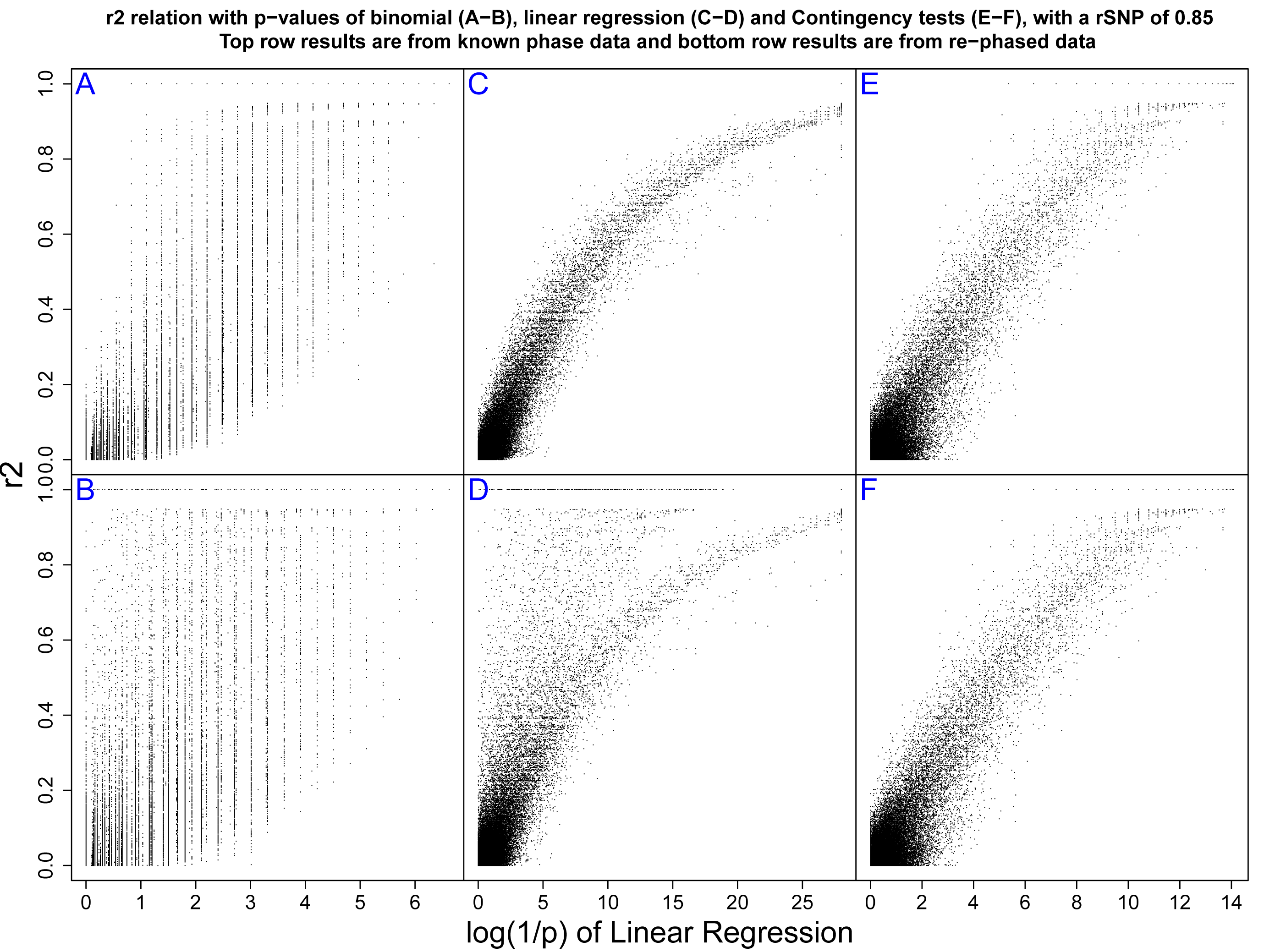

Supplement: Figure S3 — Extent of linkage disequilibrium and significance level. Plots of r2 coefficient between the R site and all tested SNPs and the corresponding log(1/p) from simulations at r frequency of ∼0.85, with known phase (upper panels) and after rephasing with PHASE (lower panels), for the binomial (A and B), linear regression (C and D) and contingency test (E and F). (TIF) [file pone.0038667.s003.tif]

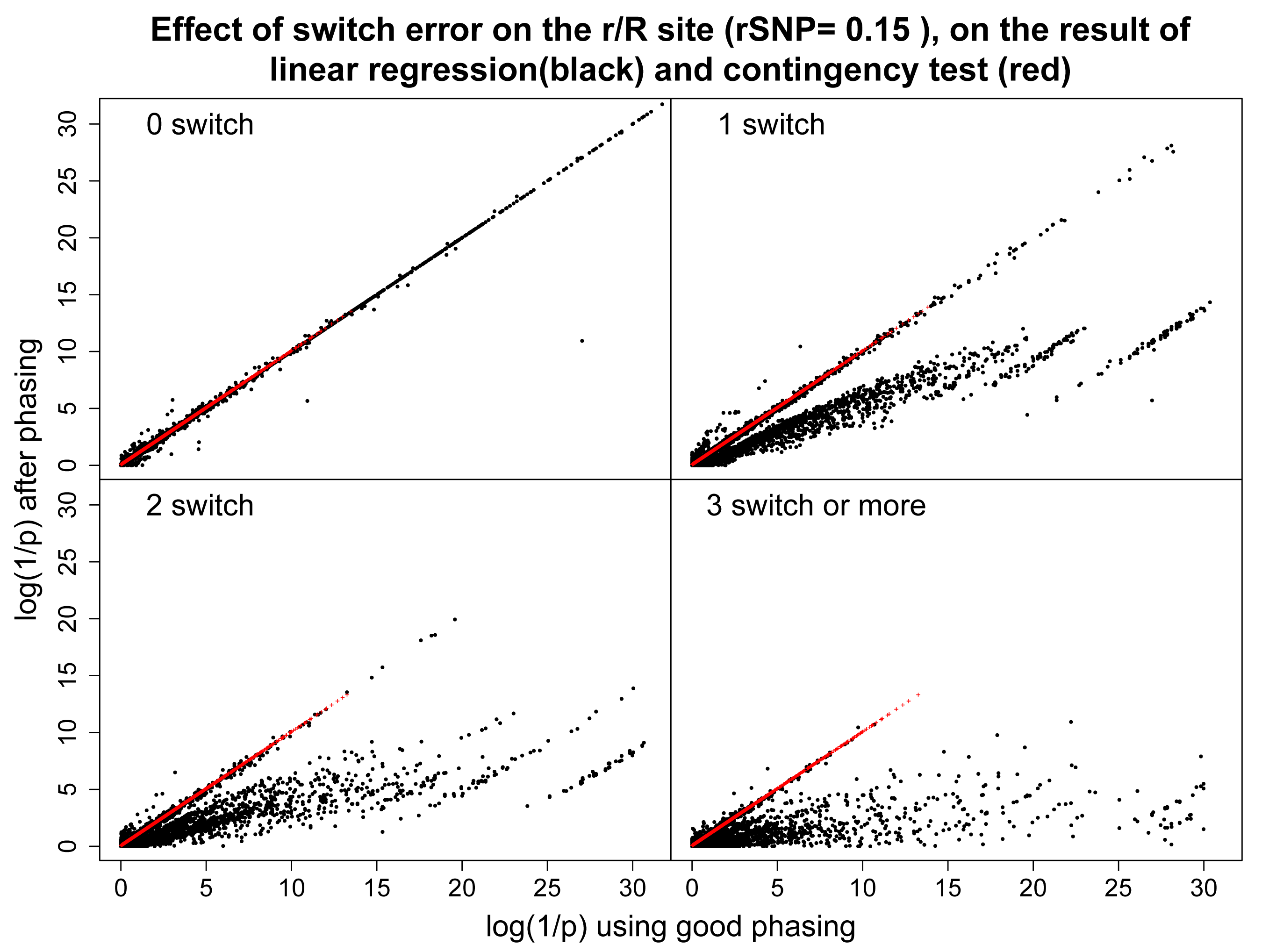

Supplement: Figure S4 — Comparison of log(1/p) values obtained before and after rephasing with PHASE. Simulations at r frequency of ∼0.15 (i.e. around 23 AI individuals out of a total of 50) were used and results were separated according to the rephasing quality evaluated as (A) zero, (B) one, (C) two and (D) three or more, AI individuals with phase inversion. (TIF) [file pone.0038667.s004.tif]

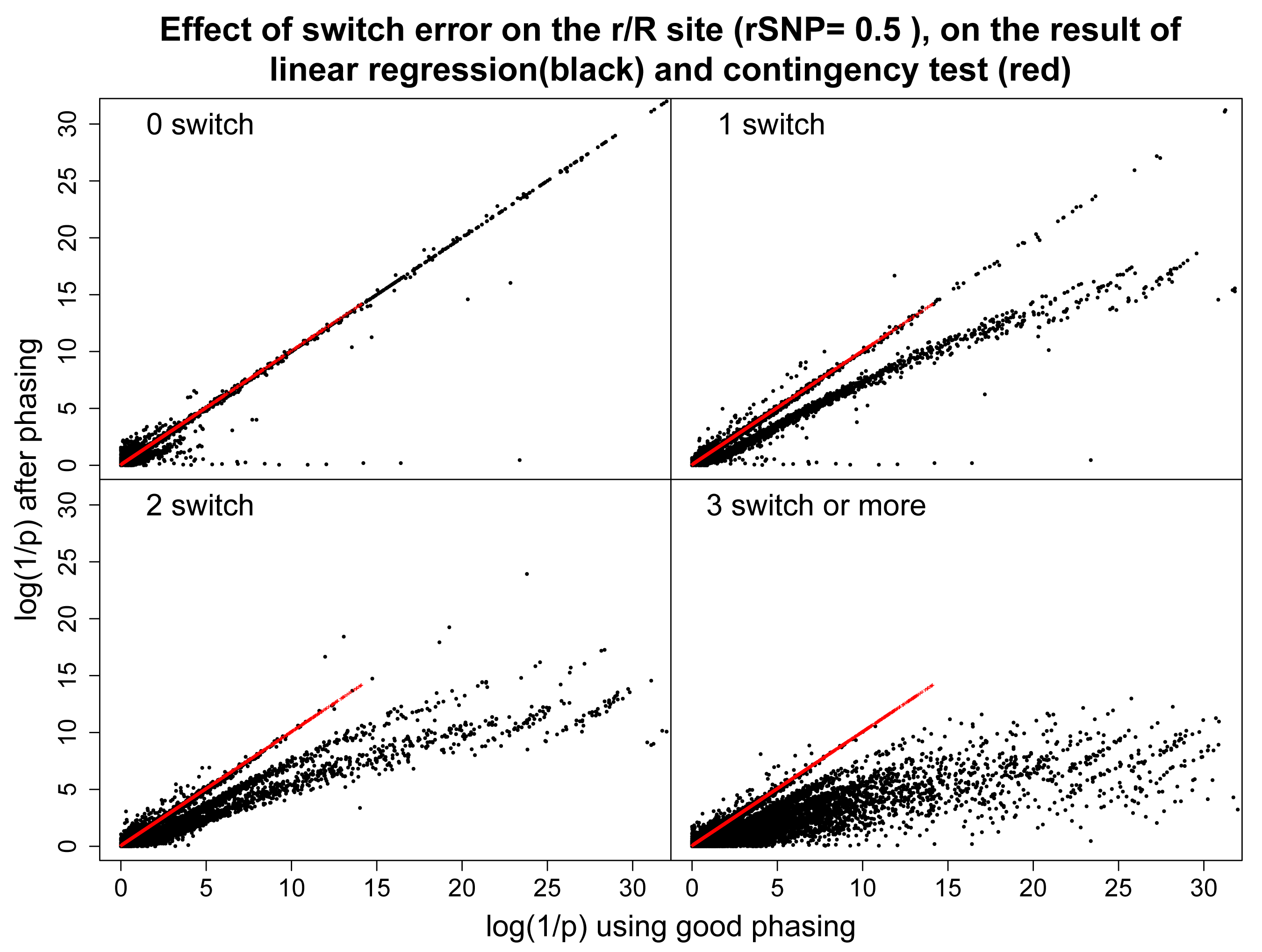

Supplement: Figure S5 — Comparison of log(1/p) values obtained before and after rephasing with PHASE. Simulations at r frequency of ∼0.5 (i.e. around 23 AI individuals out of a total of 50) were used and results were separated according to the rephasing quality evaluated as (A) zero, (B) one, (C) two and (D) three or more, AI individuals with phase inversion. (TIF) [file pone.0038667.s005.tif]

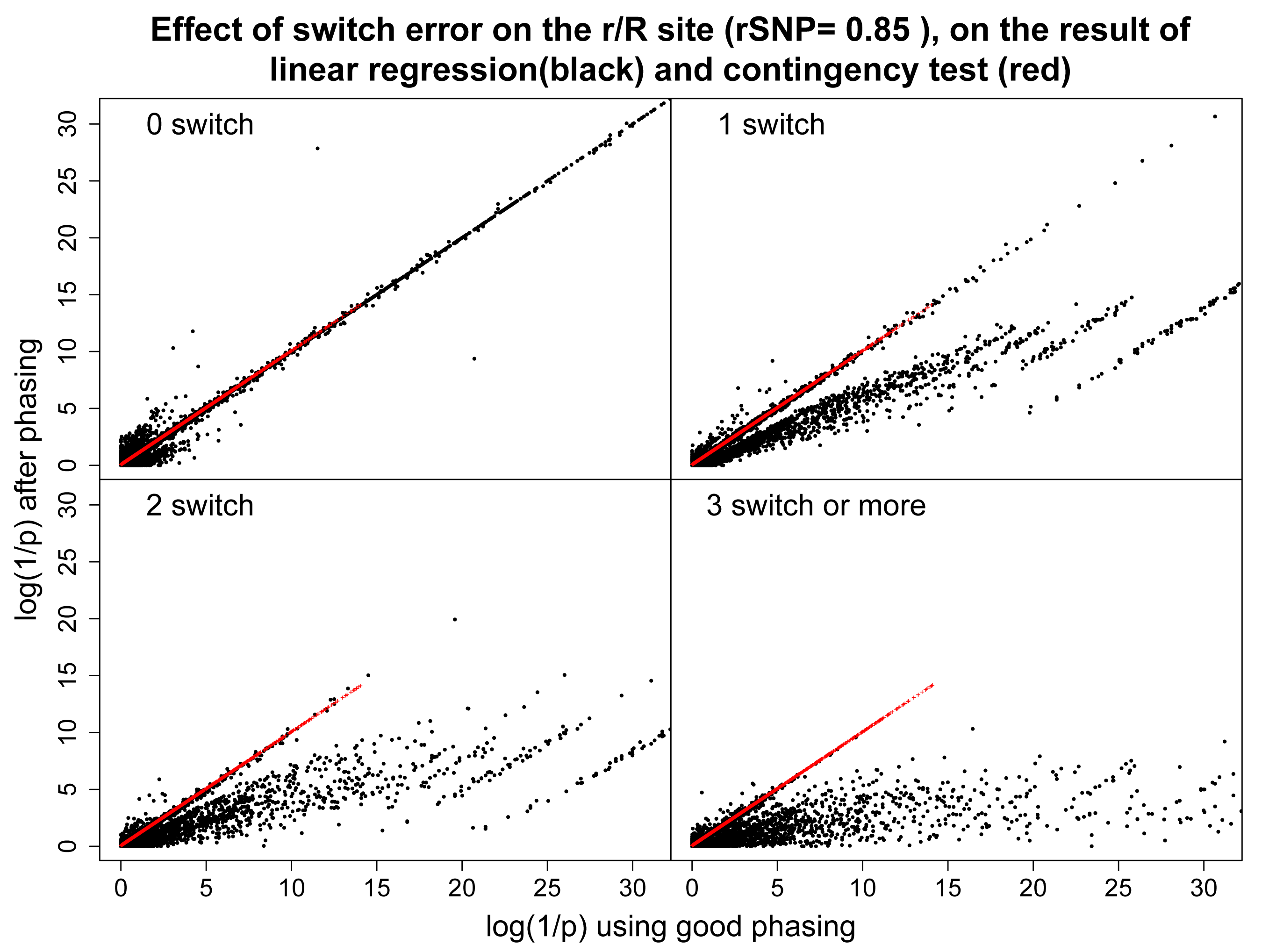

Supplement: Figure S6 — Comparison of log(1/p) values obtained before and after rephasing with PHASE. Simulations at r frequency of ∼0.85 (i.e. around 23 AI individuals out of a total of 50) were used and results were separated according to the rephasing quality evaluated as (A) zero, (B) one, (C) two and (D) three or more, AI individuals with phase inversion. (TIF) [file pone.0038667.s006.tif]

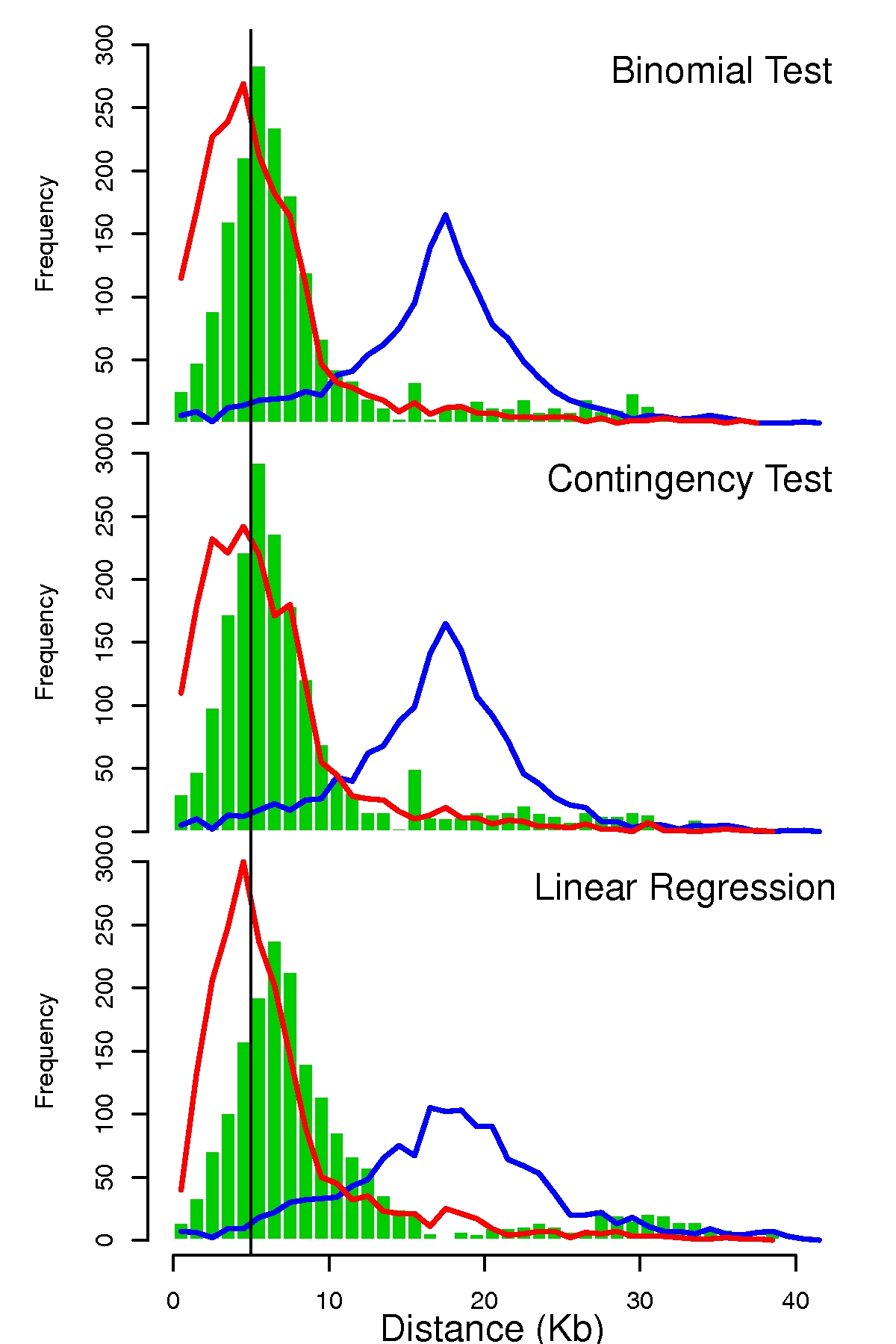

Supplement: Figure S7 — Distributions of the mean distances of the 5 lowest p -values A -sites to the regulatory SNP. Simulation results with recombination are shown by the red line (uniform) and green bars (single recombination hotspot). Those in the absence of recombination are shown by the blue line. The results from the binomial, contingency and linear regression tests are presented in downward order. (TIF) [file pone.0038667.s007.tif]

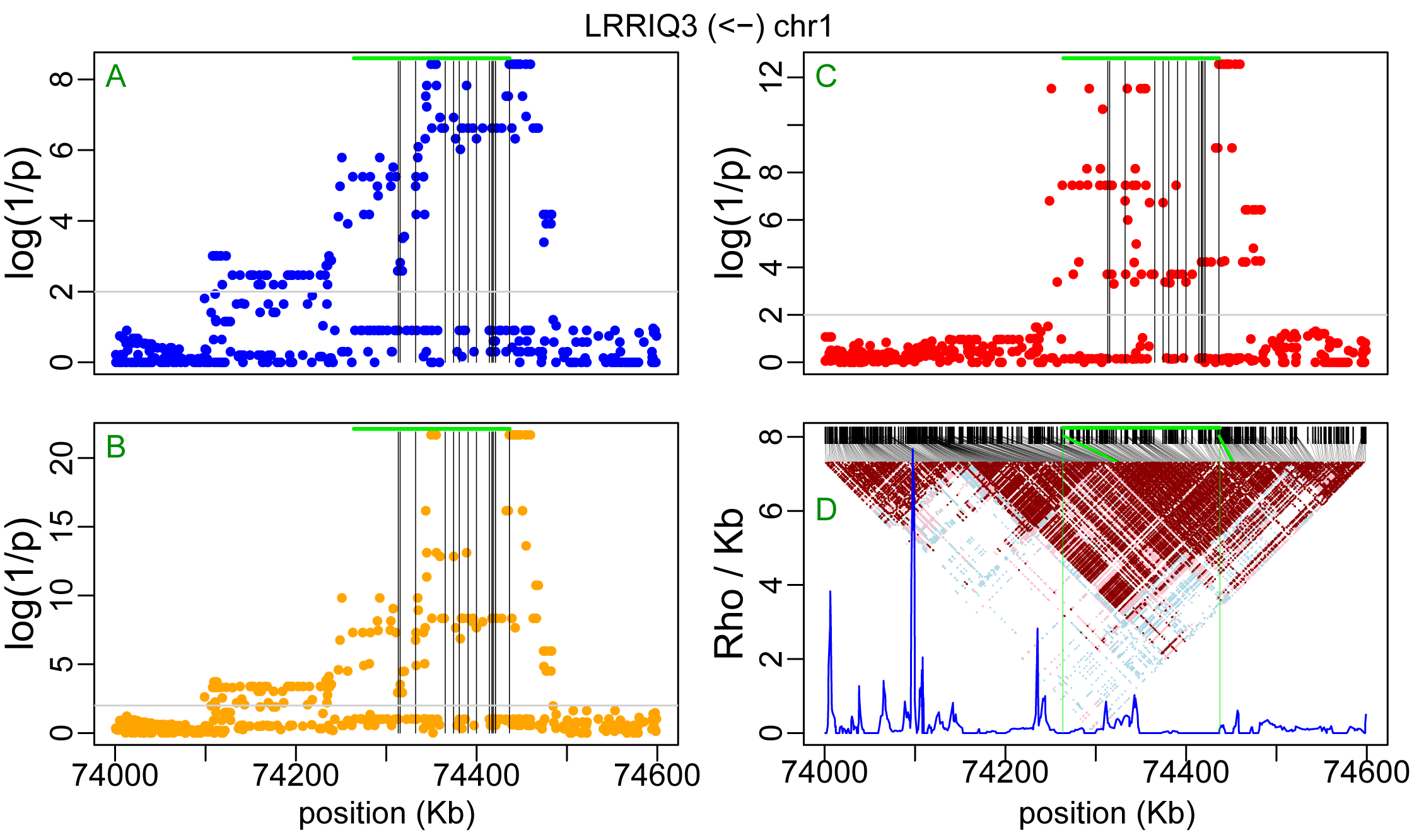

Supplement: Figure S8 — Mapping regulatory sites for LRRIQ3 (Ge, Pokholok et al. 2009). Plots of p-values for HapMap2 SNPs using binomial test (A), linear regression test (B) and contingency test (C). Vertical black lines identify SNPs that were used as informative markers within the transcript and the green horizontal line corresponds to the analyzed transcript. (D)The linkage disequilibrium triangle and recombination intensity profile of the population recombination rate (ρ/kb estimated by InfRec), where, black lines connect SNPs distributed according to sequence position (upper part) with their position in the LD triangle and vertical green lines delimit the size of the analyzed transcript. Arrow on the top indicates transcription direction. (TIF) [file pone.0038667.s008.tif]

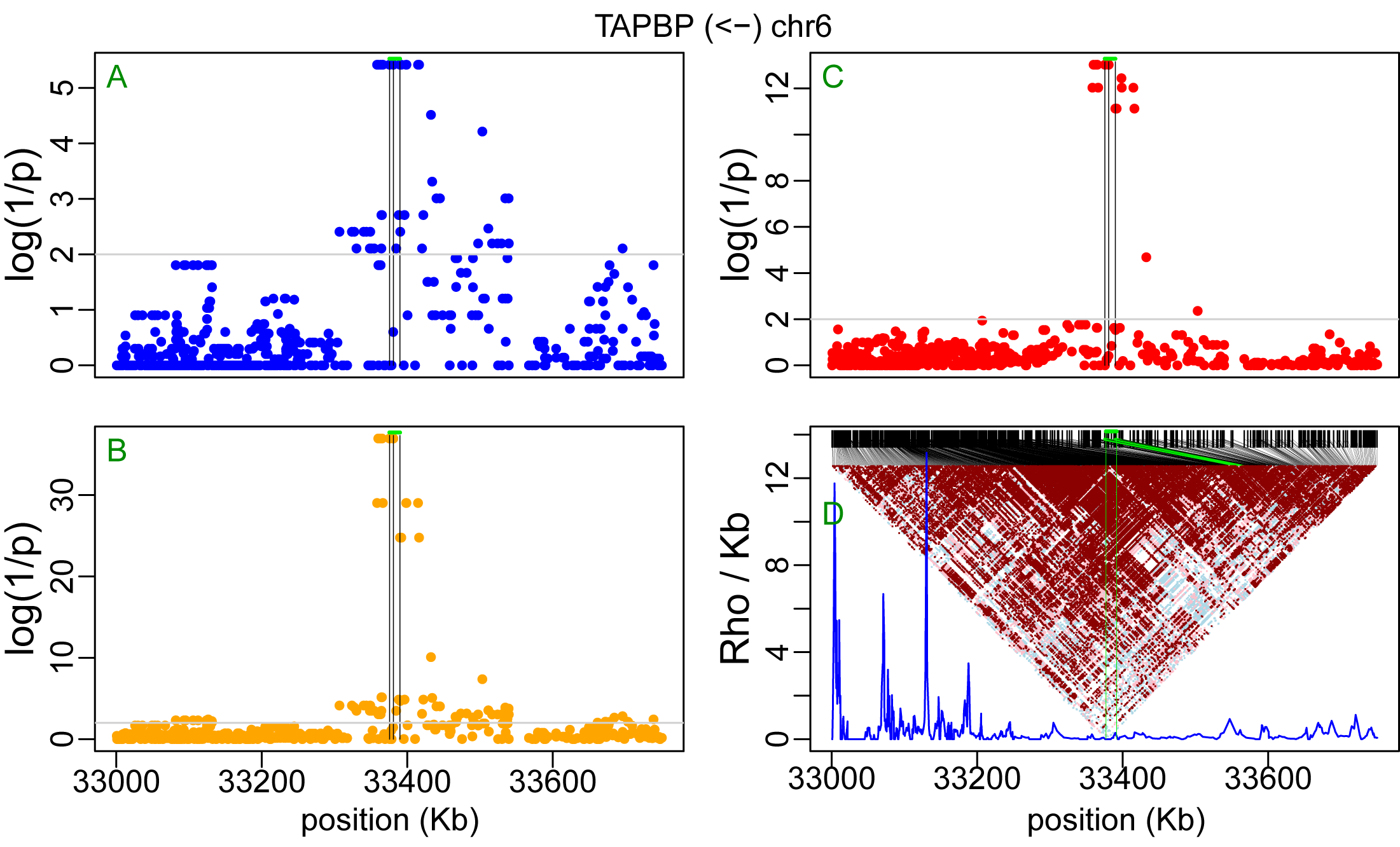

Supplement: Figure S9 — Mapping regulatory sites for TAPBP (Montgomery, Sammeth et al. 2010). Plots of p-values for HapMap3 SNPs using binomial test (A), linear regression test (B) and contingency test (C). Vertical black lines identify SNPs that were used as informative markers within the transcript and the green horizontal line corresponds to the analyzed transcript. (D) The linkage disequilibrium triangle and recombination intensity profile of the population recombination rate (ρ/kb estimated by InfRec), where, black lines connect SNPs distributed according to sequence position (upper part) with their position in the LD triangle and vertical green lines delimit the size of the analyzed transcript. Arrow on the top indicates transcription direction. (TIF) [file pone.0038667.s009.tif]

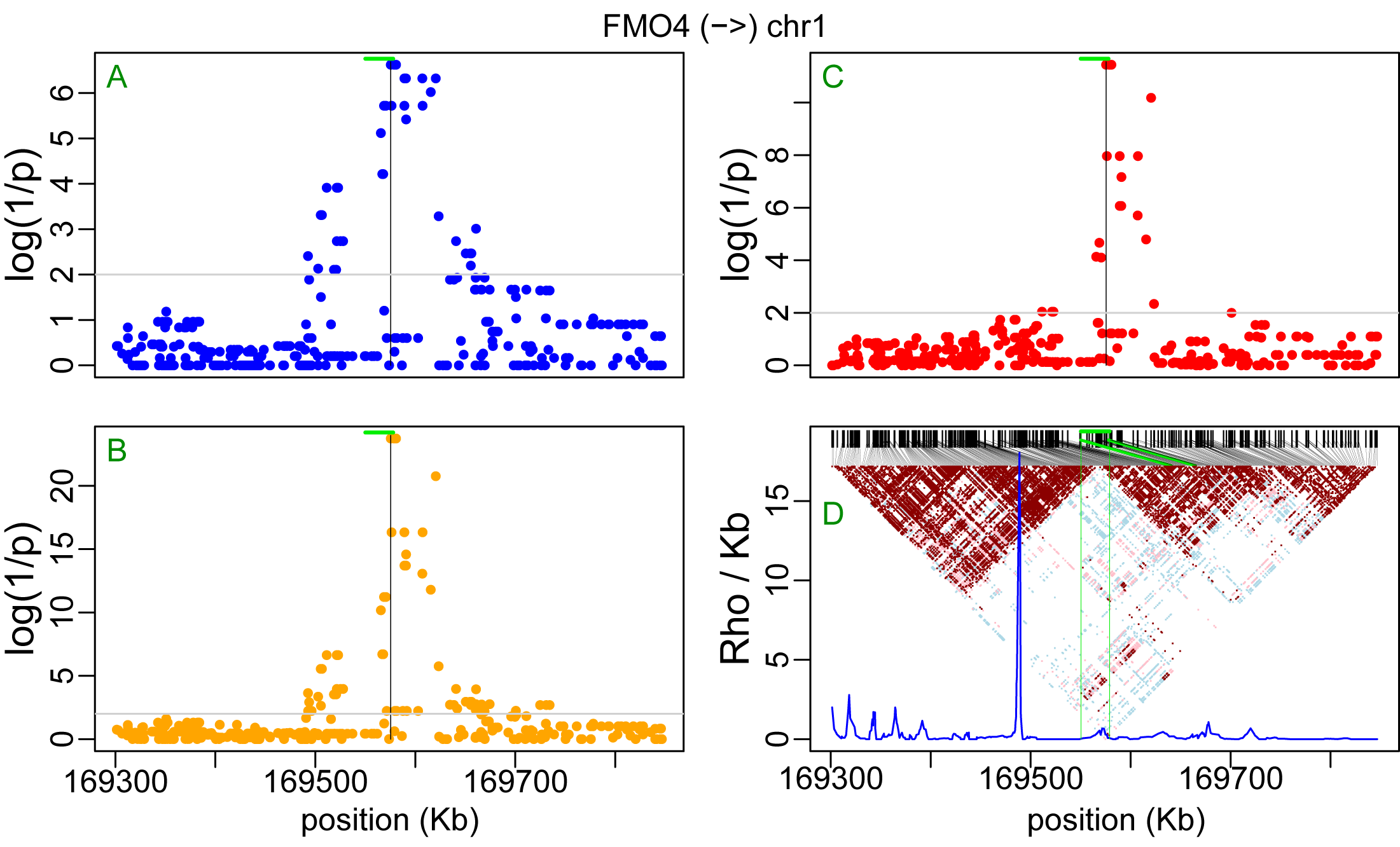

Supplement: Figure S10 — Mapping regulatory sites for FMO4 (Montgomery, Sammeth et al. 2010). Plots of p-values for HapMap3 SNPs using binomial test (A), linear regression test (B) and contingency test (C). Vertical black lines identify SNPs that were used as informative markers within the transcript and the green horizontal line corresponds to the analyzed transcript. (D) The linkage disequilibrium triangle and recombination intensity profile of the population recombination rate (ρ/kb estimated by InfRec), where, black lines connect SNPs distributed according to sequence position (upper part) with their position in the LD triangle and vertical green lines delimit the size of the analyzed transcript. Arrow on the top indicates transcription direction. (TIF) [file pone.0038667.s010.tif]

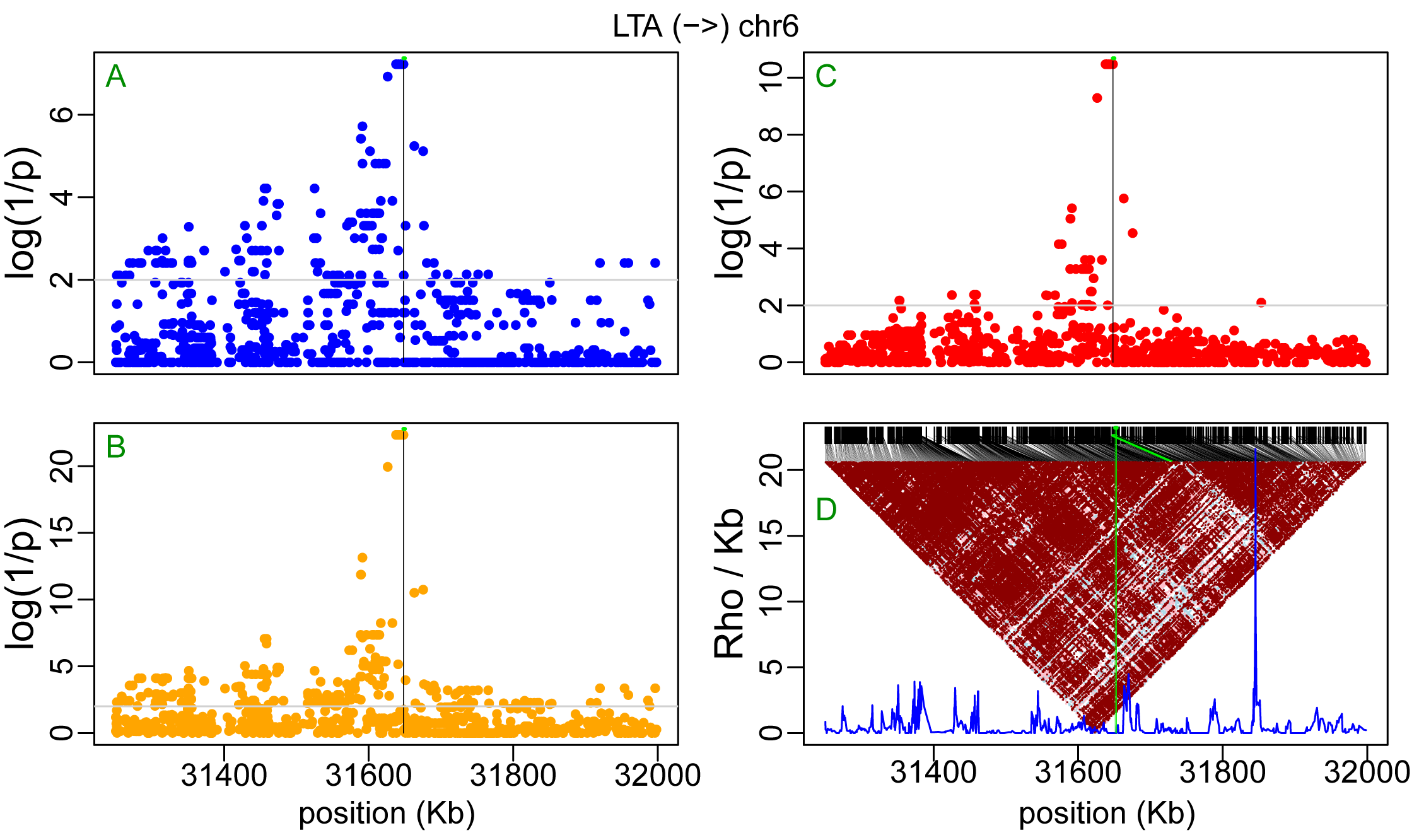

Supplement: Figure S11 — Mapping regulatory sites for LTA (Montgomery, Sammeth et al. 2010). Plots of p-values for HapMap3 SNPs using binomial test (A), linear regression test (B) and contingency test (C). Vertical black lines identify SNPs that were used as informative markers within the transcript and the green horizontal line corresponds to the analyzed transcript. (D) The linkage disequilibrium triangle and recombination intensity profile of the population recombination rate (ρ/kb estimated by InfRec), where, black lines connect SNPs distributed according to sequence position (upper part) with their position in the LD triangle and vertical green lines delimit the size of the analyzed transcript. Arrow on the top indicates transcription direction. (TIF) [file pone.0038667.s011.tif]

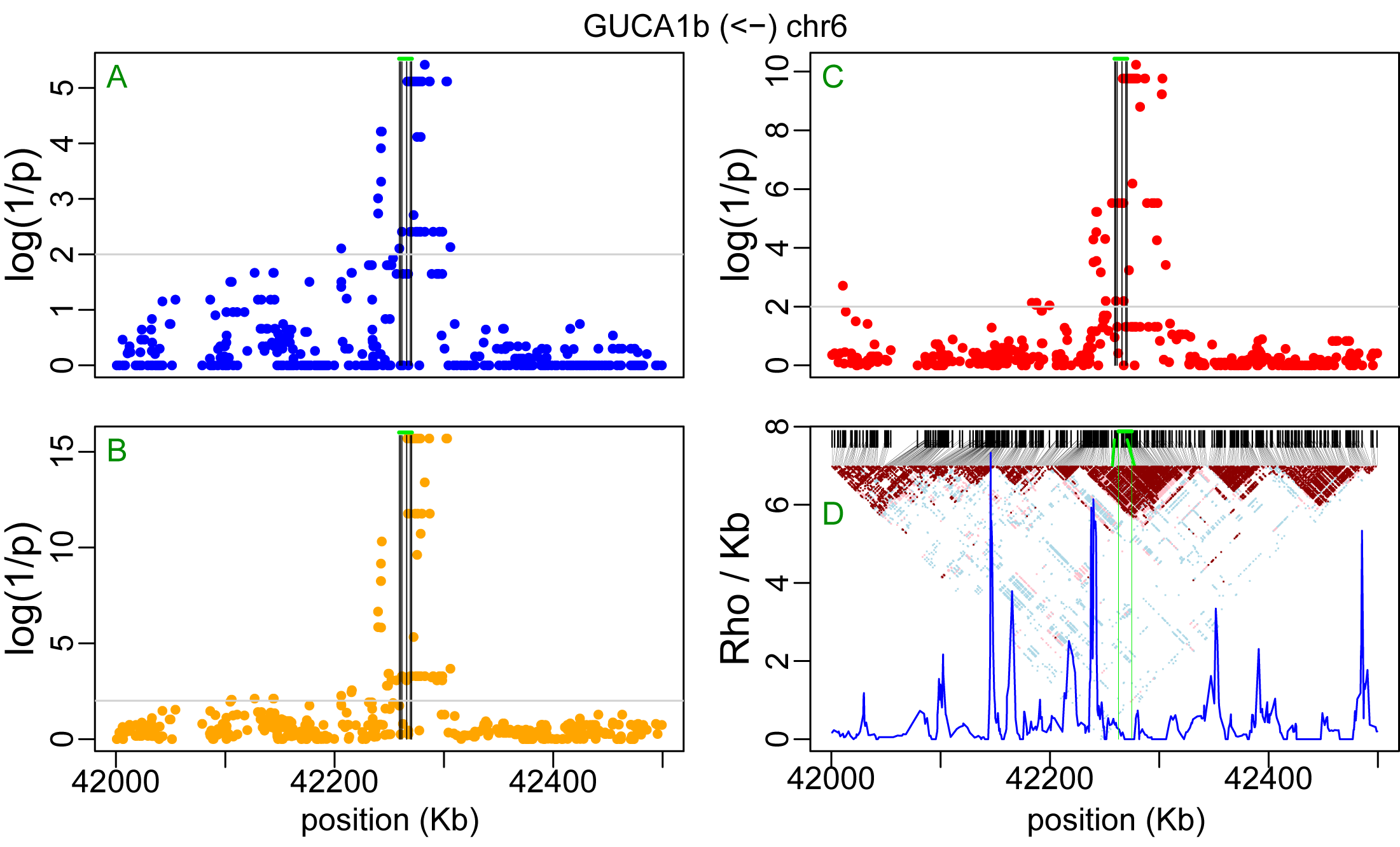

Supplement: Figure S12 — Mapping regulatory sites for GUCA (Ge, Pokholok et al. 2009). Plots of p-values for HapMap2 SNPs using binomial test (A), linear regression test (B) and contingency test (C). Vertical black lines identify SNPs that were used as informative markers within the transcript and the green horizontal line corresponds to the analyzed transcript. (D)The linkage disequilibrium triangle and recombination intensity profile of the population recombination rate (ρ/kb estimated by InfRec), where, black lines connect SNPs distributed according to sequence position (upper part) with their position in the LD triangle and vertical green lines delimit the size of the analyzed transcript. Arrow on the top indicates transcription direction. (TIF) [file pone.0038667.s012.tif]

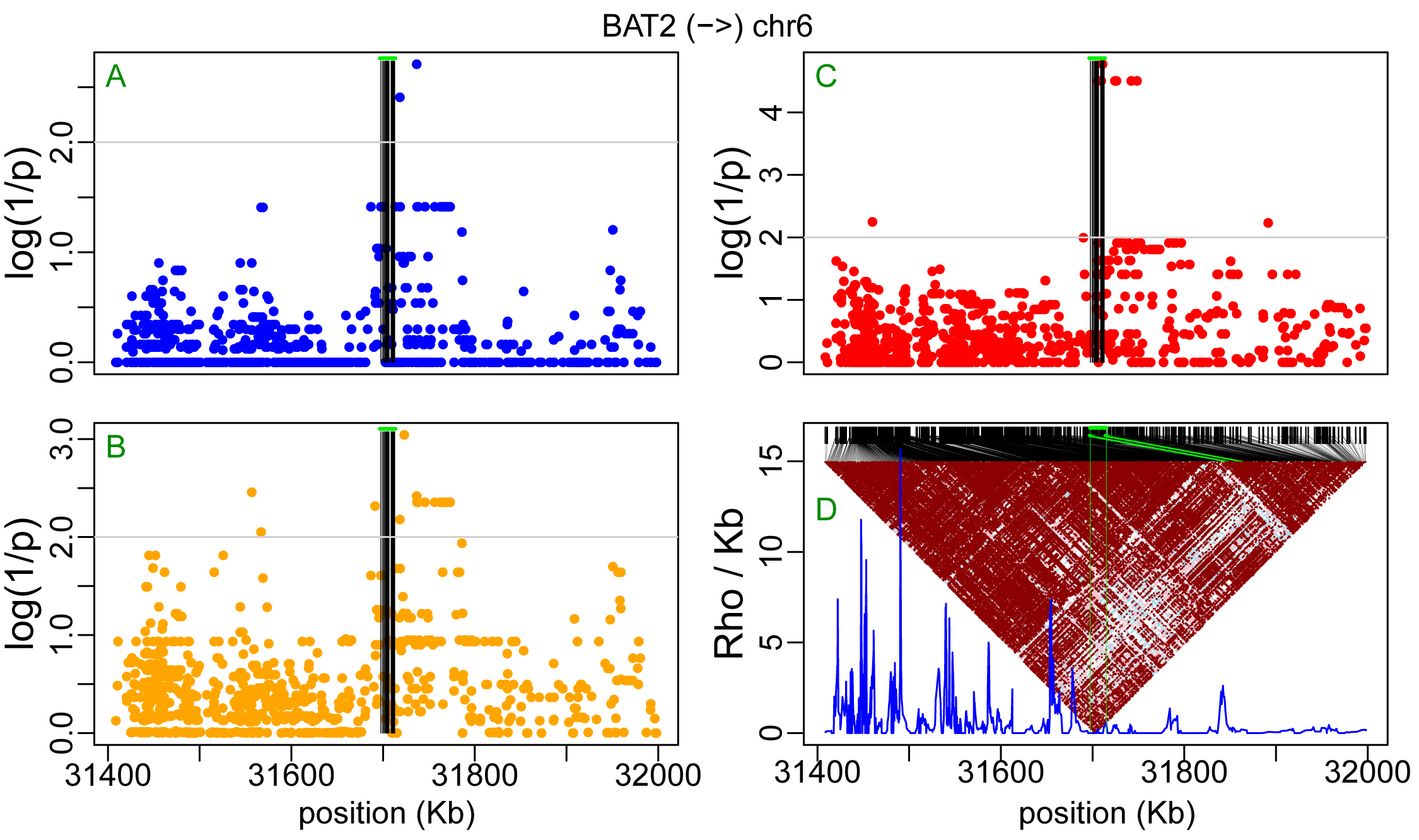

Supplement: Figure S13 — Mapping regulatory sites for BAT2 (Ge, Pokholok et al. 2009). Plots of p-values for HapMap2 SNPs using binomial test (A), linear regression test (B) and contingency test (C). Vertical black lines identify SNPs that were used as informative markers within the transcript and the green horizontal line corresponds to the analyzed transcript. (D)The linkage disequilibrium triangle and recombination intensity profile of the population recombination rate (ρ/kb estimated by InfRec), where, black lines connect SNPs distributed according to sequence position (upper part) with their position in the LD triangle and vertical green lines delimit the size of the analyzed transcript. Arrow on the top indicates transcription direction. (TIF) [file pone.0038667.s013.tif]

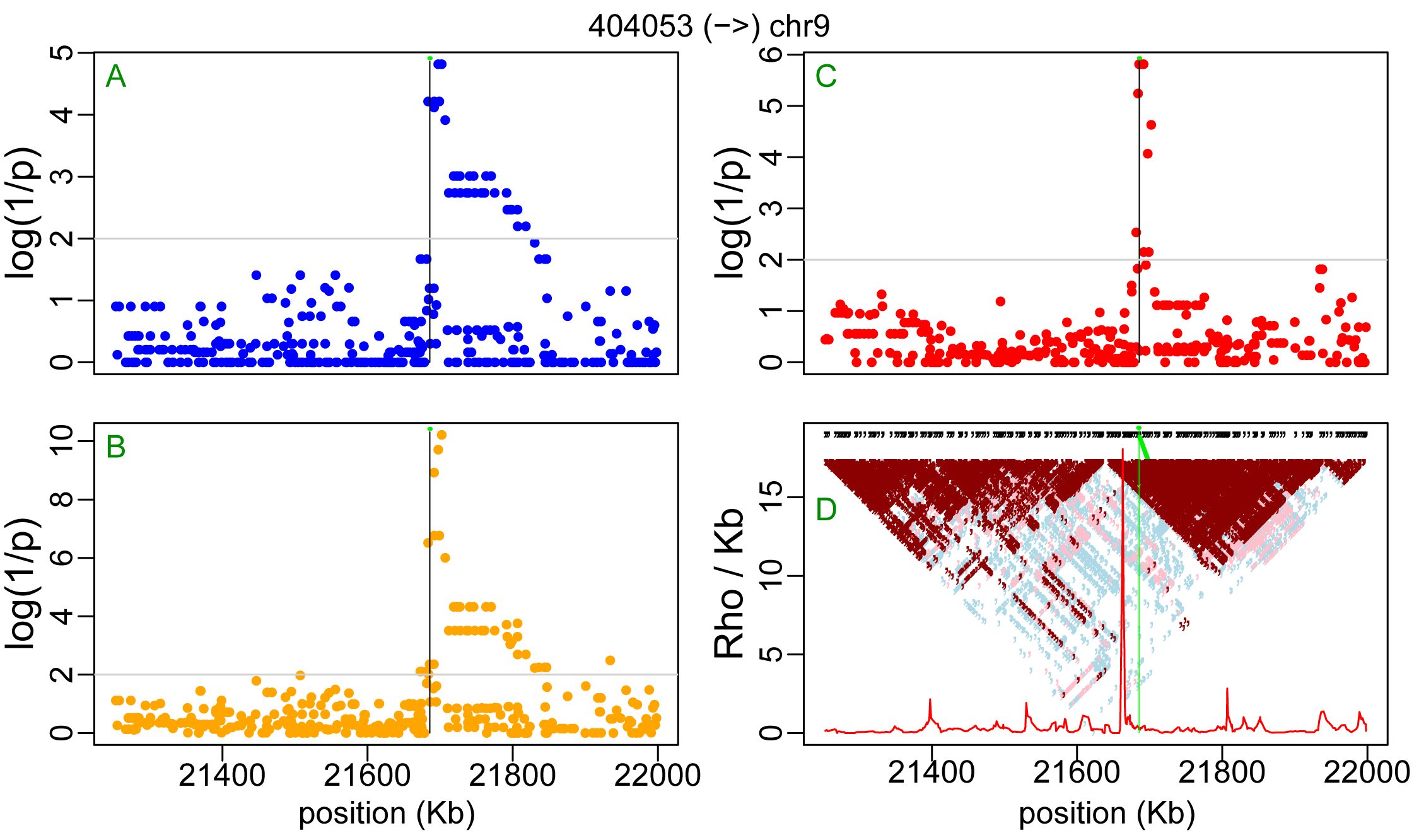

Supplement: Figure S14 — Mapping regulatory sites for transcript 404053 (Montgomery, Sammeth et al. 2010). Plots of p-values for HapMap3 SNPs using binomial test (A), linear regression test (B) and contingency test (C). Vertical black lines identify SNPs that were used as informative markers within the transcript and the green horizontal line corresponds to the analyzed transcript. (D) The linkage disequilibrium triangle and recombination intensity profile of the population recombination rate (ρ/kb estimated by InfRec), where, black lines connect SNPs distributed according to sequence position (upper part) with their position in the LD triangle and vertical green lines delimit the size of the analyzed transcript. Arrow on the top indicates transcription direction. (TIF) [file pone.0038667.s014.tif]

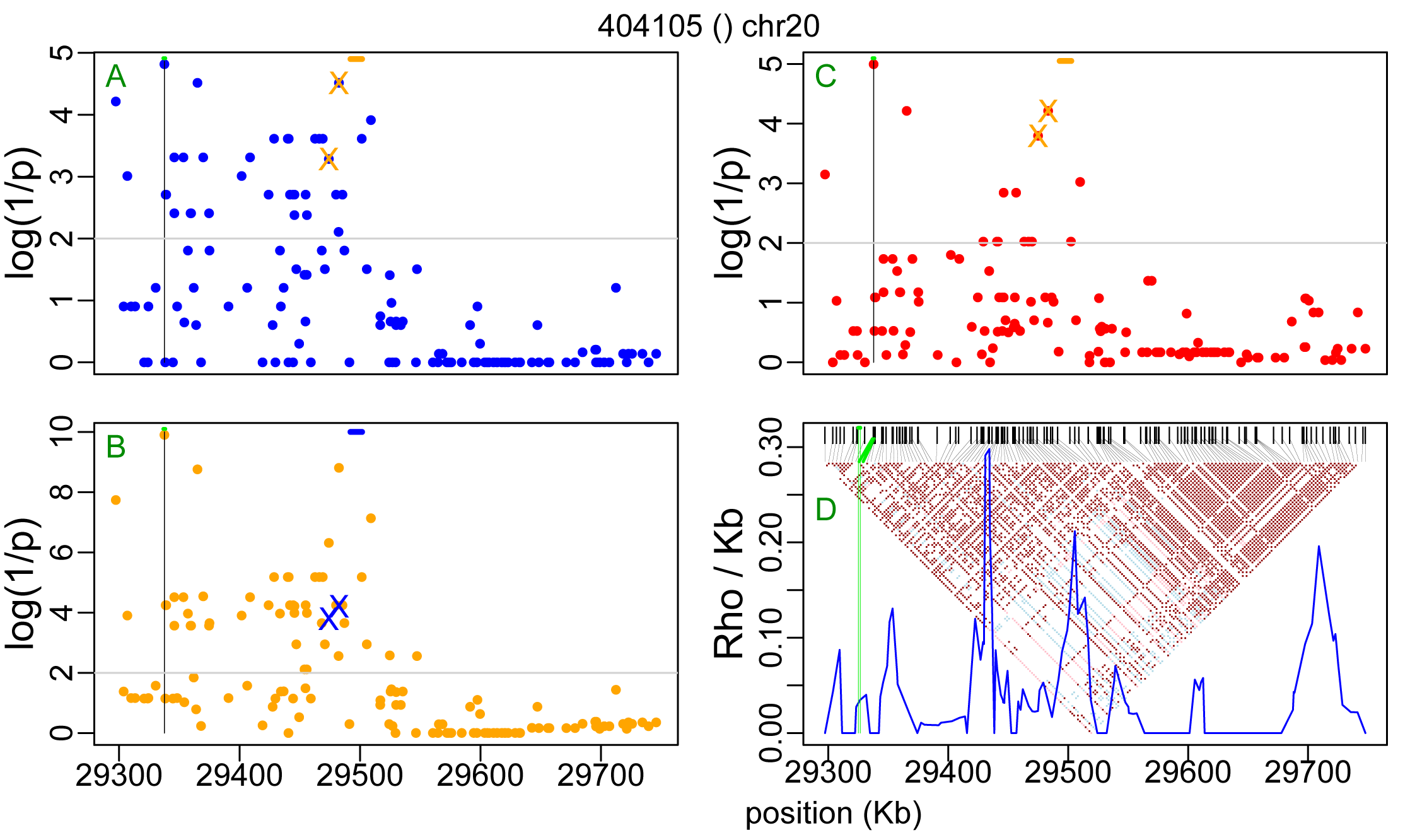

Supplement: Figure S15 — Mapping regulatory sites for transcript 404105 (Montgomery, Sammeth et al. 2010). Plots of p-values for HapMap3 SNPs using binomial test (A), linear regression test (B) and contingency test (C). Vertical black lines identify SNPs that were used as informative markers within the transcript and the green horizontal line corresponds to the analyzed transcript. Arrow on the top indicates transcription direction. Two crossed sites represent SNPs that were identified in the GWAS on late-onset Alzheimer disease: rs2180566 in the DEFB123 (orange line) promoter and rs6059244 more to the left (see Table 3). (D) The linkage disequilibrium triangle and recombination intensity profile of the population recombination rate (ρ/kb estimated by InfRec), where, black lines connect SNPs distributed according to sequence position (upper part) with their position in the LD triangle and vertical green lines delimit the size of the analyzed transcript. (TIF) [file pone.0038667.s015.tif]

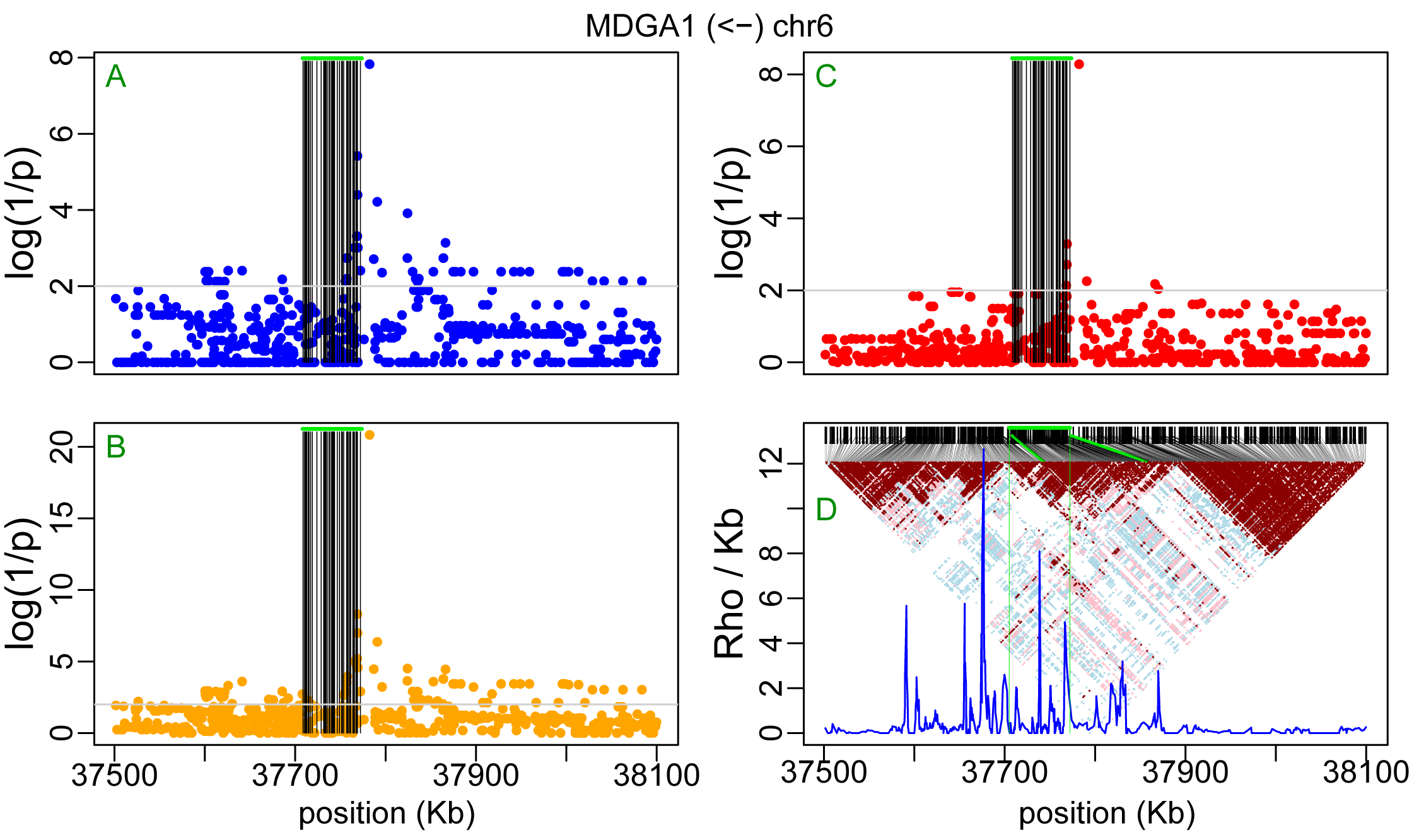

Supplement: Figure S16 — Mapping regulatory sites for MDGA1 (Ge, Pokholok et al. 2009). Plots of p-values for HapMap2 SNPs using binomial test (A), linear regression test (B) and contingency test (C). Vertical black lines identify SNPs that were used as informative markers within the transcript and the green horizontal line corresponds to the analyzed transcript. (D)The linkage disequilibrium triangle and recombination intensity profile of the population recombination rate (ρ/kb estimated by InfRec), where, black lines connect SNPs distributed according to sequence position (upper part) with their position in the LD triangle and vertical green lines delimit the size of the analyzed transcript. Arrow on the top indicates transcription direction. (TIF) [file pone.0038667.s016.tif]

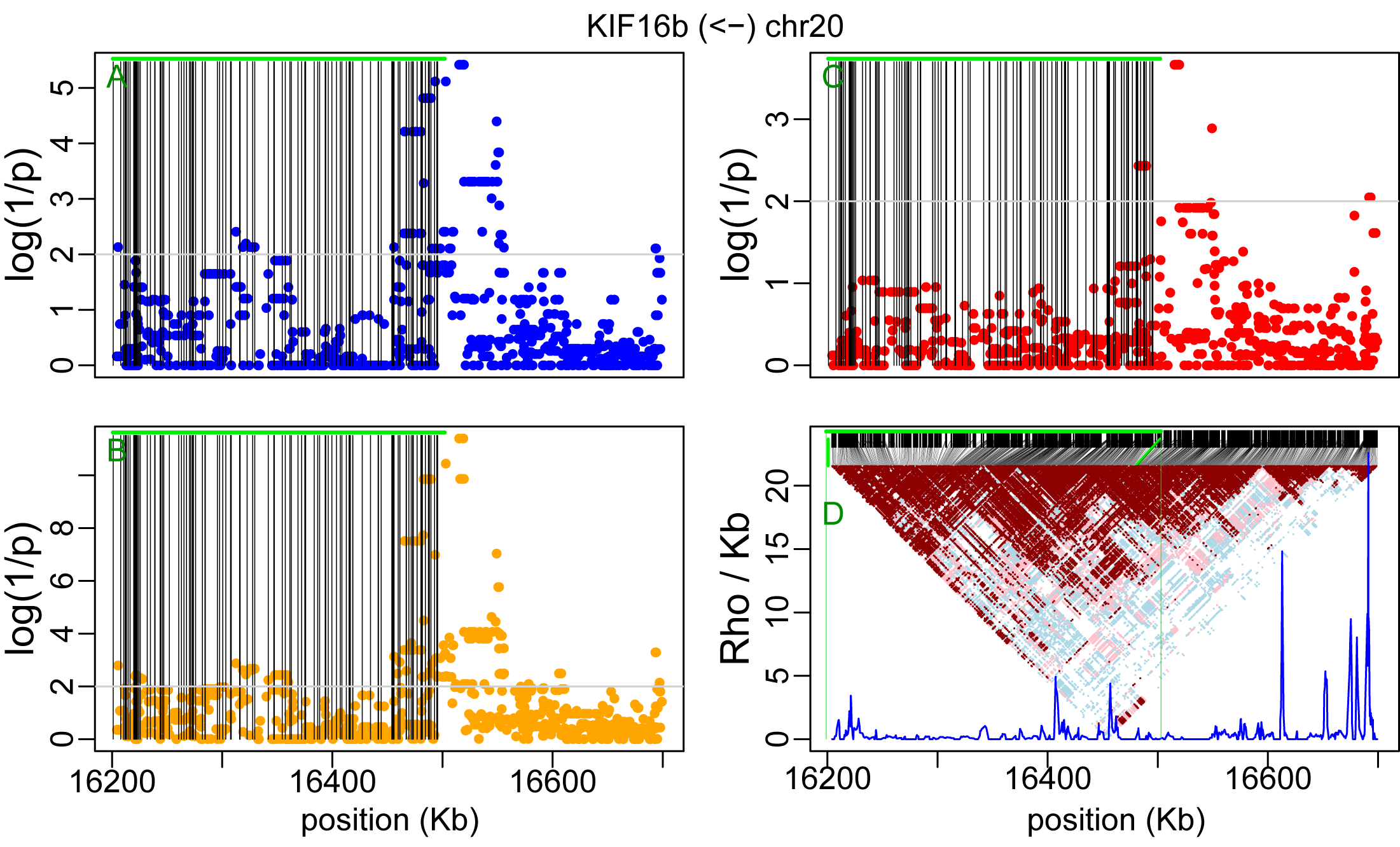

Supplement: Figure S17 — Mapping regulatory sites for KIF16b (Ge, Pokholok et al. 2009). Plots of p-values for HapMap2 SNPs using binomial test (A), linear regression test (B) and contingency test (C). Vertical black lines identify SNPs that were used as informative markers within the transcript and the green horizontal line corresponds to the analyzed transcript. (D)The linkage disequilibrium triangle and recombination intensity profile of the population recombination rate (ρ/kb estimated by InfRec), where, black lines connect SNPs distributed according to sequence position (upper part) with their position in the LD triangle and vertical green lines delimit the size of the analyzed transcript. Arrow on the top indicates transcription direction. (TIFF) [file pone.0038667.s017.tiff]

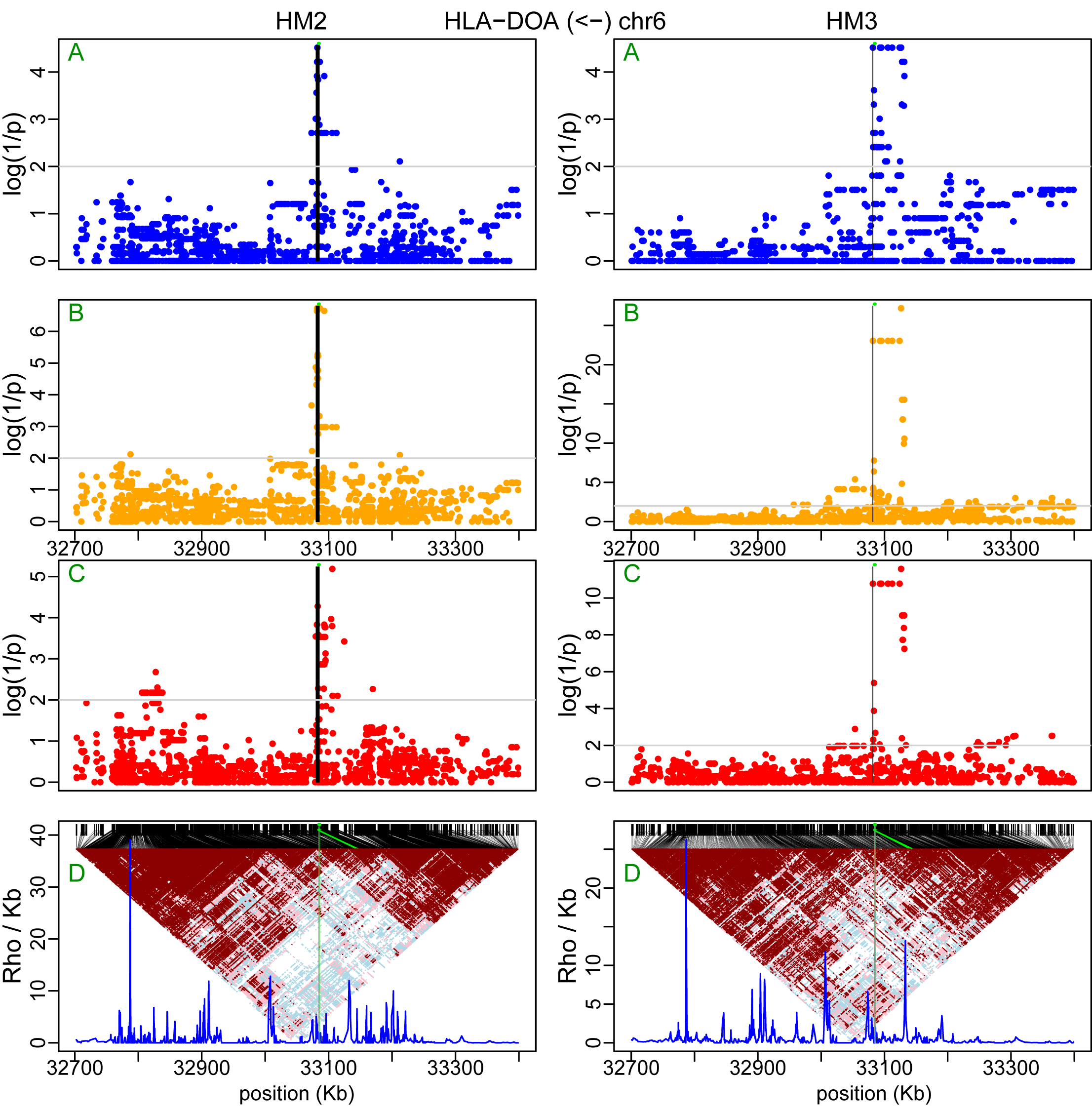

Supplement: Figure S18 — Comparison of the results from Ge, Pokholok et al. 2009 (left, based on HapMap2) with those from Montgomery, Sammeth et al. 2010 (right, based on HapMap3). Plots of p-values for HapMap2 (left) and HapMap3 (right) SNPs using binomial test (A), linear regression test (B) and contingency test (C). Vertical black lines identify SNPs that were used as informative markers within the transcript and the green horizontal line corresponds to the analyzed transcript. (D)The linkage disequilibrium triangle and recombination intensity profile of the population recombination rate (ρ/kb estimated by InfRec), where, black lines connect SNPs distributed according to sequence position (upper part) with their position in the LD triangle and vertical green lines delimit the size of the analyzed transcript. Arrow on the top indicates transcription direction. (TIFF) [file pone.0038667.s018.tiff]

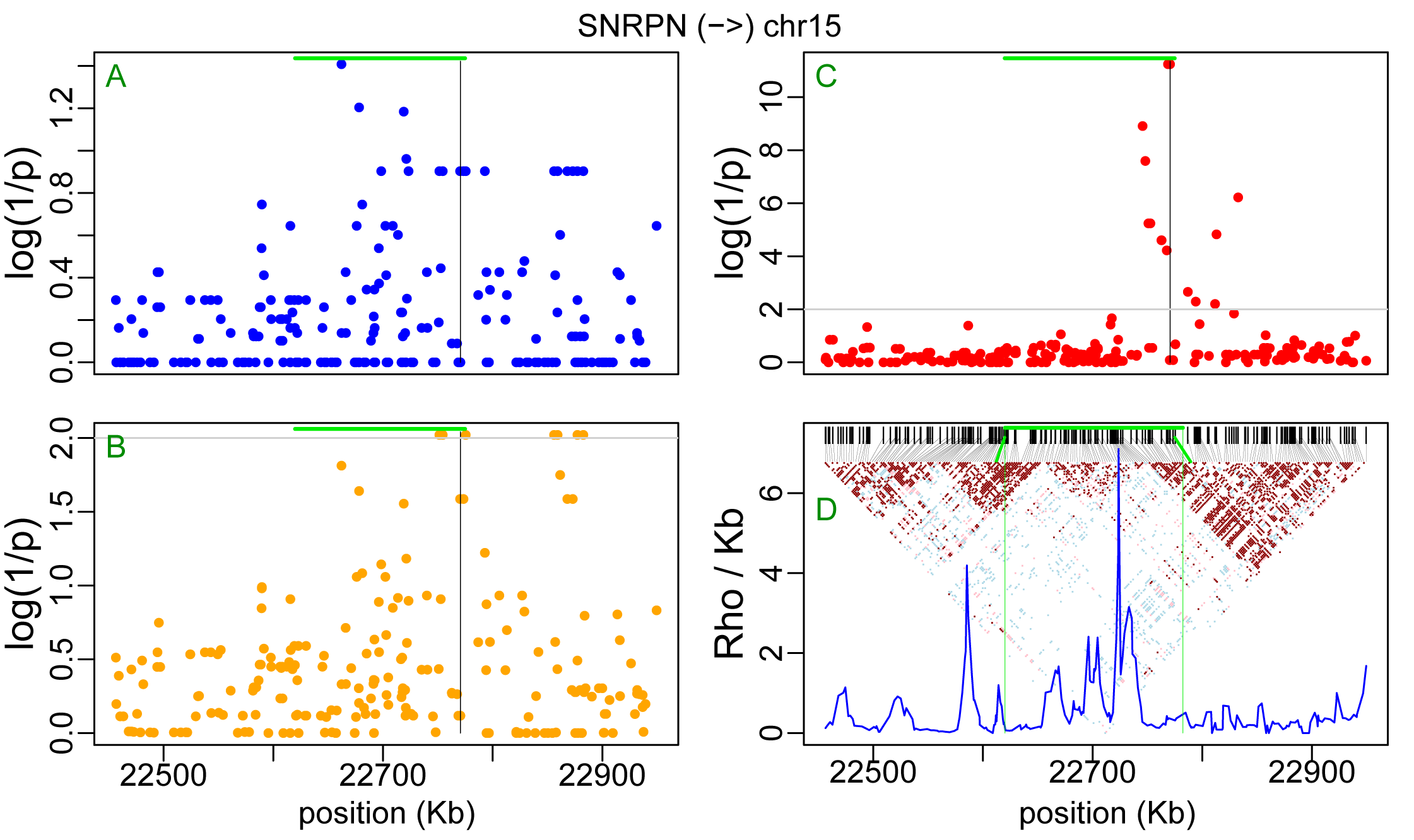

Supplement: Figure S19 — Analysis of ASE in SNRPN. Plots of p-values for HapMap3 SNPs using binomial test (A), linear regression test (B) and contingency test (C). Vertical black lines identify SNPs that were used as informative markers within the transcript and the green horizontal line corresponds to the analyzed transcript. (D) The linkage disequilibrium triangle and recombination intensity profile of the population recombination rate (ρ/kb estimated by InfRec), where, black lines connect SNPs distributed according to sequence position (upper part) with their position in the LD triangle and vertical green lines delimit the size of the analyzed transcript. Arrow on the top indicates transcription direction. (TIF) [file pone.0038667.s019.tif]

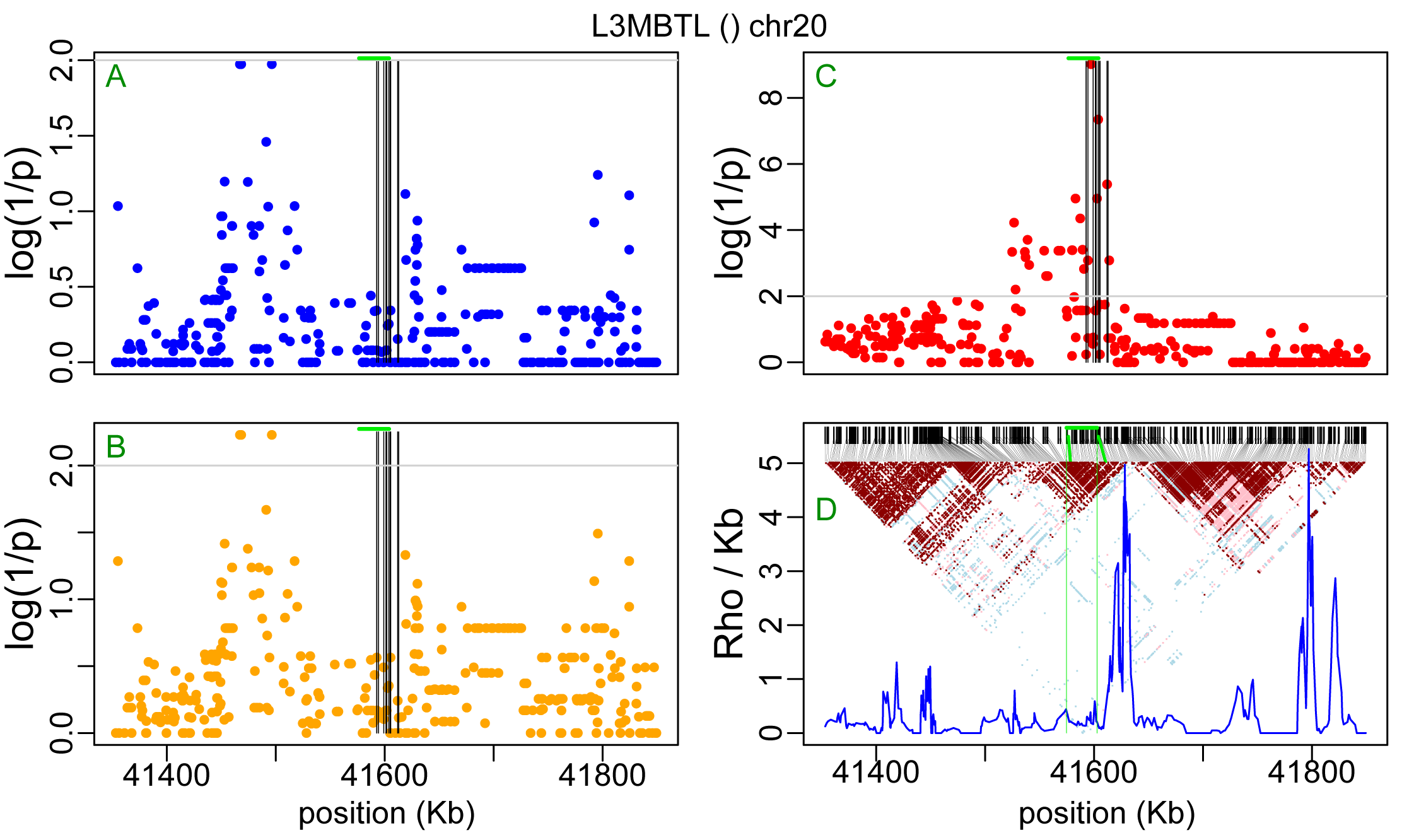

Supplement: Figure S20 — Analysis of ASE in L3MBTL. Plots of p-values for HapMap2 SNPs using binomial test (A), linear regression test (B) and contingency test (C). Vertical black lines identify SNPs that were used as informative markers within the transcript and the green horizontal line corresponds to the analyzed transcript. (D)The linkage disequilibrium triangle and recombination intensity profile of the population recombination rate (ρ/kb estimated by InfRec), where, black lines connect SNPs distributed according to sequence position (upper part) with their position in the LD triangle and vertical green lines delimit the size of the analyzed transcript. Arrow on the top indicates transcription direction. (TIF) [file pone.0038667.s020.tif]

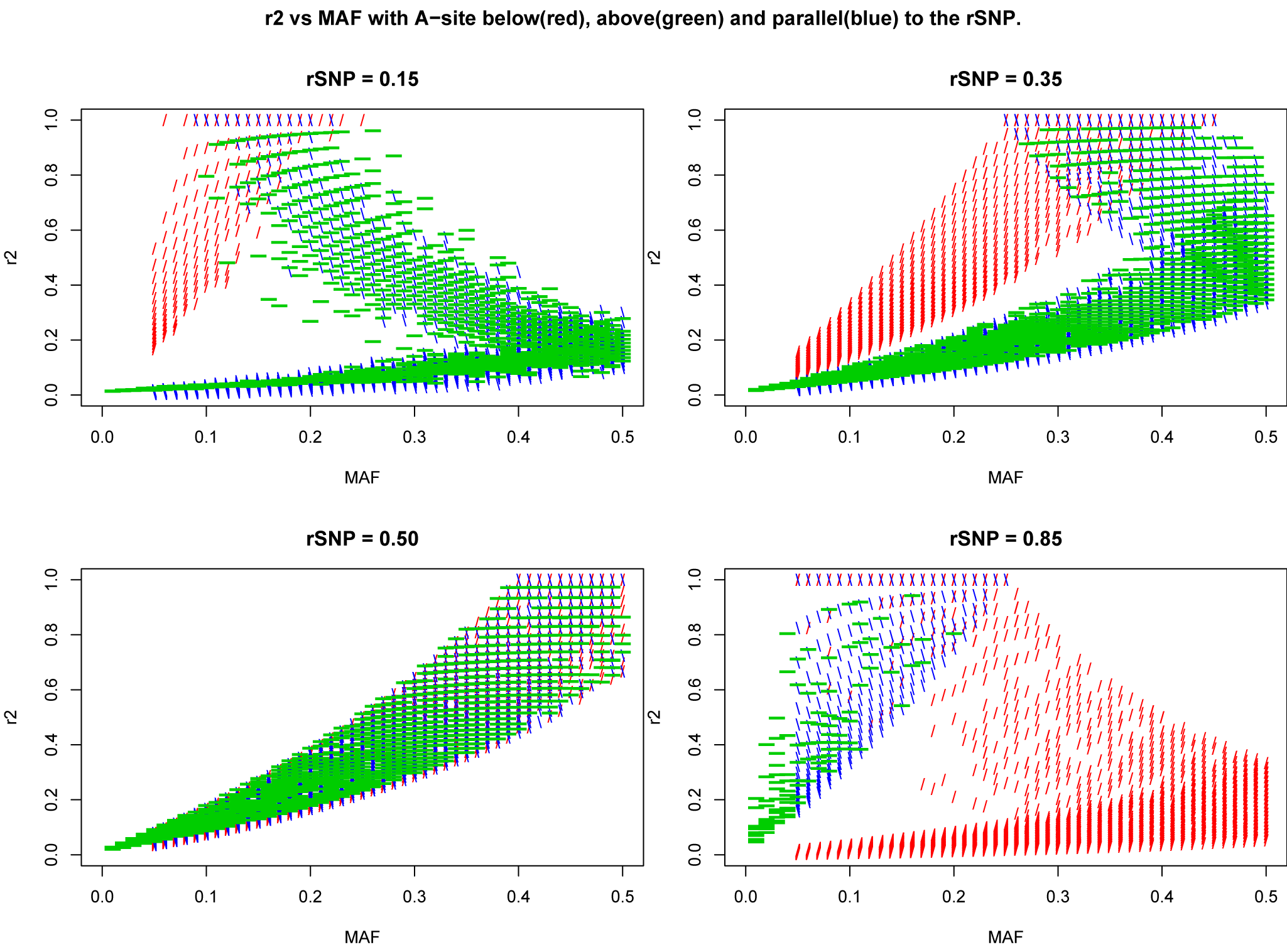

Supplement: Figure S21 — Relation between the linkage disequilibrium between the R site and all tested SNPs (r2 coefficient), and the corresponding Minor Allele Frequency (MAF). Green dash are for the SNPs above the rSNP, red slash are for below and blue back-slash are for parallel SNPs. The four different rSNP frequencies tested are shown. (TIF) [file pone.0038667.s021.tif]

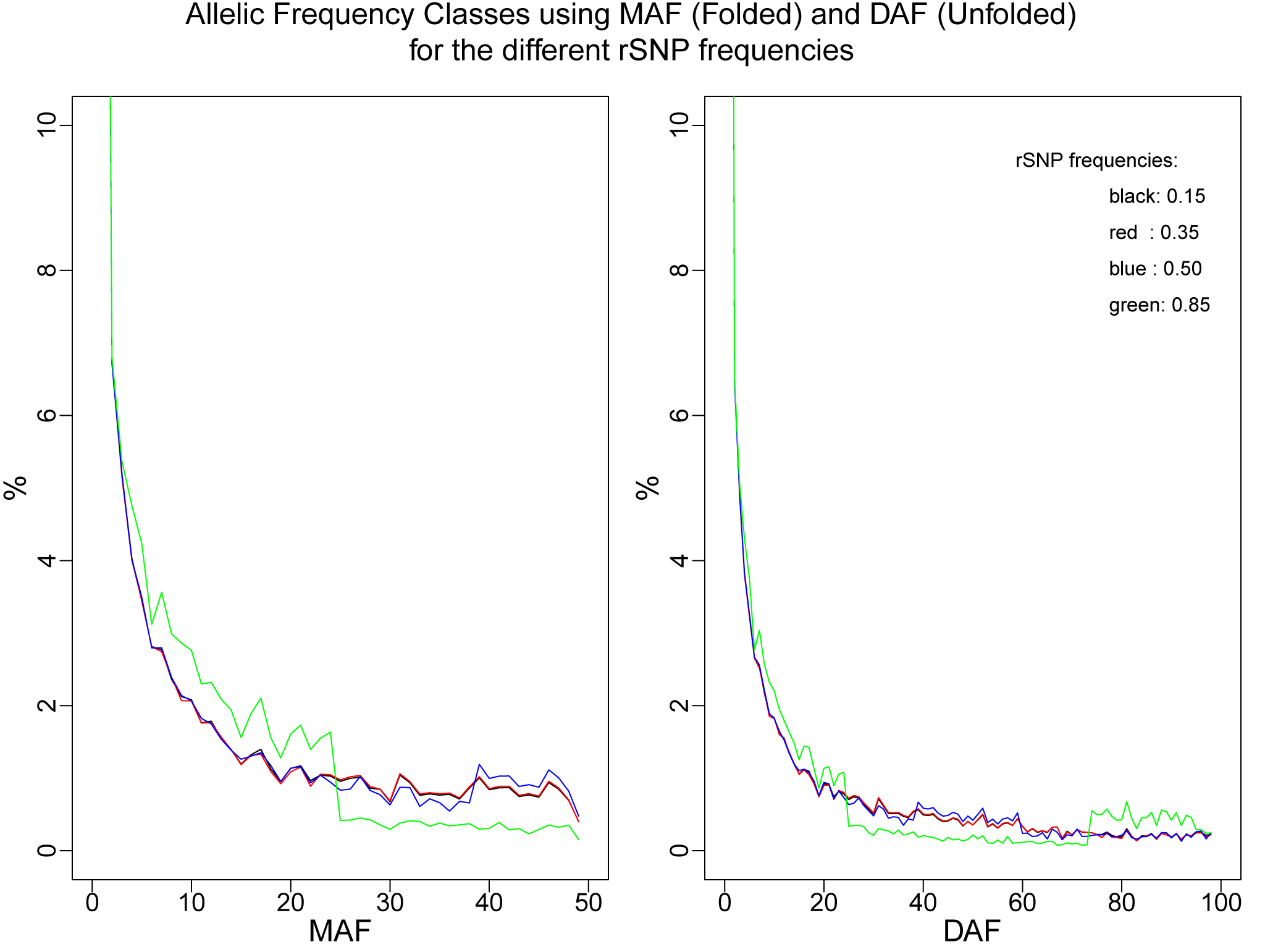

Supplement: Figure S22 — Folded and unfolded allelic frequency spectra from the different r frequencies sets of simulations. Both spectra are from four subsets of 2000 simulations where a rSNP of frequency 0.15 (black), 0.35 (red), 0.5 (blue) and 0.85 (green), could be assigned. The subsets are 2000, 1954, 1681 and 897 for 0.15, 0.35, 0.5 and 0.85, respectively. (TIF) [file pone.0038667.s022.tif]
