# Supplementary material for: Genotype-Based Test in Mapping Cis-Regulatory Variants from Allele-Specific Expression Data
Source: PLoS One. 2012 Jun 7;7(6):e38667. doi: 10.1371/journal.pone.0038667 (PMC3369843; doi:10.1371/journal.pone.0038667)
Supplement: Table S1 — Lowest and mean p-values, expressed in log(1/p), of the significant results of the three tests performed on three sets of simulations, the “no-recombination” set, the “recombination with phase known” and the “recombination with re-phased data”. (DOCX) [file pone.0038667.s023.docx]

**Table S1. Lowest and mean p-values, expressed in log(1/p), of the significant results of the three tests performed on three sets of simulations, the “no-recombination” set, the “recombination with phase known” and the “recombination with re-phased data”.**

| **No Recombination** | | | | |
| --- | --- | --- | --- | --- |
| r-frequency | 0.15 | 0.35 | 0.5 | 0.85 |
| **Max log10(1/p)** | | | | |
| Binomial | 6.62 | 9.93 | 10.84 | 7.22 |
| Contingency | 14.03 | 14.08 | 14.08 | 14.03 |
| Linear regression | 28 | 28 | 28 | 28.00 |
| **Mean log10(1/p)** | | | | |
| Binomial | 2.57 | 2.92 | 2.91 | 2.8 |
| Contingency | 2.96 | 3.08 | 3.02 | 3.46 |
| Linear regression | 2.95 | 2.99 | 3.01 | 3.42 |
| **Recombination with phase known** | | | | |
| r-frequency | 0.15 | 0.35 | 0.5 | 0.85 |
| **Max log10(1/p)** | | | | |
| Binomial | 6.02 | 9.93 | 10.54 | 7.22 |
| Contingency | 13.83 | 14.08 | 14.08 | 14.08 |
| Linear regression | 28 | 28 | 28 | 28 |
| **Mean log10(1/p)** | | | | |
| Binomial | 2.56 | 2.81 | 2.82 | 2.71 |
| Contingency | 2.84 | 2.9 | 2.88 | 3.06 |
| Linear regression | 2.81 | 2.86 | 2.87 | 3.04 |
| **Recombination with re-phased data** | | | | |
| r-frequency | 0.15 | 0.35 | 0.5 | 0.85 |
| **Max log10(1/p)** | | | | |
| Binomial | 5.42 | 8.73 | 9.63 | 6.62 |
| Contingency | 13.83 | 14.08 | 14.08 | 14.08 |
| Linear regression | 28 | 28 | 28 | 28 |
| **Mean log10(1/p)** | | | | |
| Binomial | 2.45 | 2.68 | 2.69 | 2.6 |
| Contingency | 2.84 | 2.9 | 2.88 | 3.06 |
| Linear regression | 2.77 | 2.79 | 2.79 | 3.00 |

Note: The lowest p-value becomes the highest log(1/p). The significant results are p<0.01. For programming purposes, the lower limit of the p-values was set to 1e-28.
