# Supplementary material for: Genotype-Based Test in Mapping Cis-Regulatory Variants from Allele-Specific Expression Data
Source: PLoS One. 2012 Jun 7;7(6):e38667. doi: 10.1371/journal.pone.0038667 (PMC3369843; doi:10.1371/journal.pone.0038667)
Supplement: Table S2 — Distribution of segregating sites across genealogical trees preselected to carry r alleles above certain frequency threshold and its effect on r2, derived allele frequency (DAF) and power of the tests in each site position category. (DOCX) [file pone.0038667.s024.docx]

**Table S2. Distribution of segregating sites across genealogical trees preselected to carry r alleles above certain frequency threshold and its effect on r^2^, derived allele frequency (DAF) and power of the tests in each site position category.**

| r-frequencies | 0.15 | 0.35 | 0.5 | 0.85 |
| --- | --- | --- | --- | --- |
| Fraction of the 2000 simulated genealogies that is compatible with the r-frequency | | | | |
|  | 2000 | 1954 | 1681 | 897 |
| Repartition of genealogical positions of *A*-sites relative to *R* (%) | | | | |
| below | 9.1 | 20.9 | 28.3 | 63.6 |
| parallel | 67.5 | 61.8 | 55.5 | 33.6 |
| above | 23.4 | 17.3 | 16.2 | 2.9 |
| Mean r^2^ observed within each position category | | | | |
| below | 0.83 | 0.59 | 0.43 | 0.29 |
| parallel | 0.09 | 0.21 | 0.28 | 0.82 |
| above | 0.20 | 0.27 | 0.33 | 0.32 |
| total | 0.19 | 0.30 | 0.34 | 0.47 |
| Mean DAF observed within each position category | | | | |
| below | 0.12 | 0.21 | 0.25 | 0.37 |
| parallel | 0.24 | 0.22 | 0.19 | 0.13 |
| above | 0.58 | 0.71 | 0.77 | 0.95 |
| total | 0.31 | 0.31 | 0.31 | 0.31 |
| Power of the contingency test within each position category (%) | | | | |
| below | 97.2 | 62.7 | 39.7 | 30.6 |
| parallel | 13.1 | 20.4 | 24.1 | 95.2 |
| above | 39.1 | 34.6 | 35.8 | 45.2 |
| total | 26.8 | 32.2 | 30.9 | 52.7 |
| Power of the binomial test within each position category (%) | | | | |
| below | 75.8 | 68.6 | 57.4 | 28.3 |
| parallel | 13.0 | 35.1 | 49.8 | 68.5 |
| above | 37.8 | 54.8 | 66.0 | 15.6 |
| total | 24.9 | 45.8 | 54.8 | 41.5 |
| Power of the linear regression test within each position category (%) | | | | |
| below | 99.8 | 87.4 | 68.6 | 37.4 |
| parallel | 20.5 | 42.0 | 63.8 | 99.1 |
| above | 49.6 | 59.4 | 74.2 | 72.7 |
| total | 34.8 | 54.8 | 67.0 | 59.1 |

Only sites with MAF 5% were considered in the analysis. Note that with the increasing frequency of r-allele, a number of simulations are eliminated because certain tree topologies cannot accommodate sites with a required derived allele frequency.
